# Downregulated miR-150-5p in the Tissue of Nasopharyngeal Carcinoma

**DOI:** 10.1155/2022/2485055

**Published:** 2022-09-05

**Authors:** Jia-Ying Wen, Gang Chen, Jian-Di Li, Jia-Yuan Luo, Juan He, Ren-Sheng Wang, Li-Ting Qin

**Affiliations:** ^1^Department of Radiotherapy, The First Affiliated Hospital of Guangxi Medical University, No 6 Shuangyong Rd, Nanning 530021, Guangxi Zhuang Autonomous Region, China; ^2^Department of Pathology, The First Affiliated Hospital of Guangxi Medical University, No 6 Shuangyong Rd, Nanning 530021, Guangxi Zhuang Autonomous Region, China

## Abstract

The clinical significance and potential targets of miR-150-5p have not been elucidated in nasopharyngeal carcinoma (NPC). The pooled analysis based on 539 NPC samples and 75 non-NPC nasopharyngeal samples demonstrated that the expression of miR-150-5p was down-regulated in NPC, with the area under the curve being 0.89 and the standardized mean difference being −0.66. Subsequently, we further screened the differentially expressed genes (DEGs) of 14 datasets, including 312 NPC samples and 70 non-NPC nasopharyngeal samples. After the DEGs were narrowed down with the predicted targets from the miRWalk database, 1316 prospective target genes of miR-150-5p were identified. The enrichment analysis suggested that “pathways in cancer” was the most significant pathway. Finally, six hub genes of “pathways in cancer”, including EGFR, TP53, HRAS, CCND1, CDH1, and FGF2, were screened out through the STRING database. In conclusion, the down-regulation of miR-150-5p modulates the tumorigenesis and progression of NPC.

## 1. Introduction

Nasopharyngeal carcinoma (NPC) is a common head and neck malignant tumor [1, 2]. In 2018, there were about 72,987 patients who died from NPC and 129,079 new cases of NPC worldwide, most of which were distributed in Southeast Asia [3]. The preferred treatment for early-stage NPC is intensity-modulated radiotherapy [4–6]. However, more than 80% of patients are locally advanced during initial diagnosis [7, 8]. For these locally advanced patients, the treatment of choice is platinum-based concurrent chemoradiotherapy. Nonetheless, 5%–15% NPC patients still developed nasopharyngeal or local lymph node recurrence after synchronous radiotherapy, and 15%–30% NPC patients developed distant metastasis [9–12]. Therefore, exploring the pathogenesis and searching for novel molecules are imperative for the diagnosis and individualized treatment of NPC.

MicroRNA (miRNA) is a class type of noncoding RNA consisting of 17–23 nucleotides [13]. miRNA has an important effect on the differentiation, migration, proliferation, and apoptosis of cancer cell by binding to 3′-UTR and regulating translation of target genes [14]. MiR-150-5p is abnormally expressed in the non-small cell lung carcinoma, rectal carcinoma, cervical carcinoma, and other carcinomas, and is closely related to their tumorigenesis and development [15–17]. However, the mechanism of miR-150-5p in NPC remains unclear.

In order to explore the role of miR-150-5p in NPC, miRNA microarrays and sequencing data of NPC were collected from online databases, and the miR-150-5p expression in NPC was determined. Subsequently, the potential target genes were obtained by combining the differentially expressed genes (DEGs) of NPC with the genes predicted using miRWalk. In addition, the DAVID database was used to identify the potential pathways and mechanisms from two aspects: Kyoto Encyclopedia of Genes and Genomes (KEGG) and Gene Ontology (GO). Finally, Cytoscape software and the STRING database were used to screen the hub genes.

## 2. Materials and Methods

### 2.1. Data Mining and Processing of the miR-150-5p

All miRNA microarrays and sequencing datasets were gathered from high-throughput databases, such as ArrayExpress, Oncomine, Sequence Read Archive, and Gene Expression Omnibus (GEO). “MicroRNA” and “NPC” were used to construct a search formula for the screening. All included datasets should meet the following standards: (1) miR-150-5p expression data must be provided, and (2) the species must be Homo sapiens. The data exclusion criteria were that (1) studies using fewer than three samples were excluded, and (2) animal-based studies were excluded. The unstandardized miRNA expression data were subjected to log2 conversion, and the retrieval deadline is of 30 May 2021.

### 2.2. Comprehensive Analysis of miR-150-5p Expression

R software 3.6.2, GraphPad Prism 8.0.2, and Stata 16.0 were used for the statistical analysis and graphing. The violin diagrams were used to visualize the expression of miR-150-5p. To measure the ability of miR-150-5p to distinguish NPC from non-NPC nasopharyngeal tissues, we generated and calculated the receiver operating characteristic (ROC) curves, the area under the curves (AUC), and the summary ROC (sROC) curve. Next, the expression of miR-150-5p in NPC and non-NPC nasopharyngeal tissues was assessed using the standardized mean difference (SMD) calculated by Stata. When SMD < 0 and 95% confidence interval (CI) < 0, miR-150-5p expression was considered to be down-regulated in NPC; otherwise, miR-150-5p expression was considered to be up-regulated in NPC. A fixed effect model was selected when *I*^2^ < 50%; otherwise, we selected a random effect model. In order to detect the publication bias, the Begg's test was used. The *P* value with statistical significance was less than 0.05.

### 2.3. Screening of Differentially Expressed Genes

All available mRNA high-throughput datasets were screened to comprehensively construct the DEG profile of NPC. Retrieval strategies and data processing were similar to those described above. The sva package was applied to merge the datasets from the same platform and remove the batch effects. And the limma package was adopted to screen the DEGs. The screening criteria were: *P* < 0.05 and |log2 fold change| > 1. Finally, SMDs were calculated for all the DEGs. The DEGs with a SMD exceeding 0 and a 95% CI not crossing the 0-point coordinate line were regarded as up-regulated; otherwise, DEGs were down-regulated.

### 2.4. Identification of Target Genes

Target gene prediction databases can use many different computational models to predict the genes that bind to miRNAs. The target genes of miR-150-5p were predicted using miRwalk. The predicted genes were further narrowed down in accordance with their expression levels in NPC. The up-regulated DEGs and the genes predicted by miRwalk were intersected using the Venn diagrams. The co-existing genes were considered as the most possible target genes of miR-150-5p.

### 2.5. The Enrichment Analysis of the Target Genes

DAVID 6.8 was used to identify potential pathways and mechanisms based on two aspects: KEGG and GO. The GO analysis contained cellular component (CC), molecular function (MF), and biological process (BP). The signaling pathways of the target genes were explored using KEGG analysis. The *P* value of the terms with statistical significance was less than 0.05.

### 2.6. Establishment of the Interaction Network

The protein–protein interaction (PPI) network was established using the STRING online public database. The score of the PPI network exceeded 0.40. Subsequently, the results from the STRING database were imported into Cytoscape 3.8.0 and further analyzed using Centiscape 2.2 to identify hub genes.

## 3. Results

### 3.1. The Expression of miR-150-5p Was Down-Regulated in NPC

**Figure 1:**
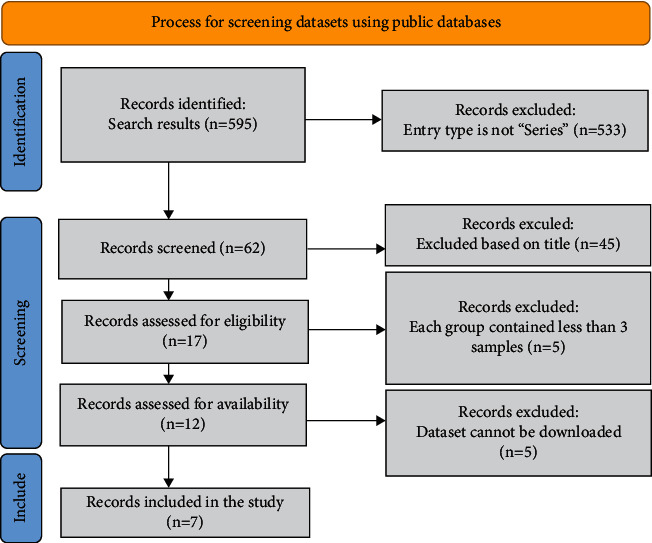
The process of miRNA datasets screening. Seven miRNA datasets are retrieved for further analysis.

**Figure 2:**
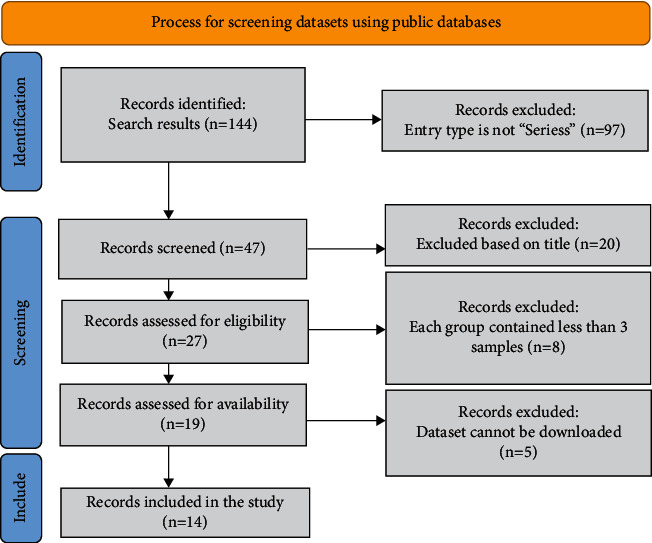
The process of mRNA screening, and fourteen mRNA datasets from the GEO database are included in this study.

**Figure 3:**
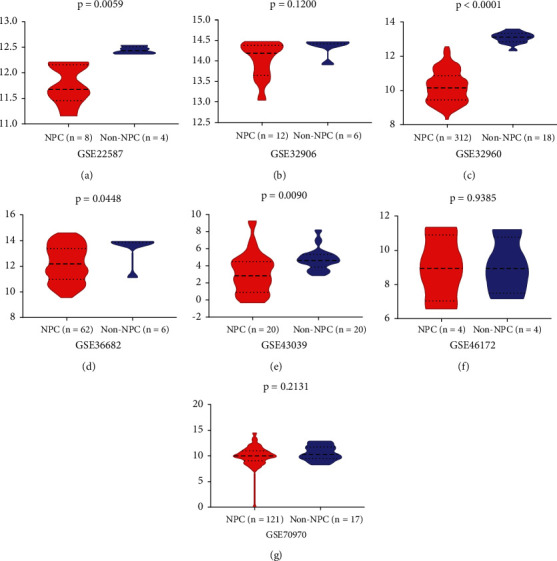
The expression of miR-150-5p in nasopharyngeal carcinoma groups and non-tumor nasopharyngeal groups. In GSE22587, GSE32960, GSE36682, and GSE43039, the expression of miR-150-5p is down-regulated in nasopharyngeal carcinoma groups.

**Figure 4:**
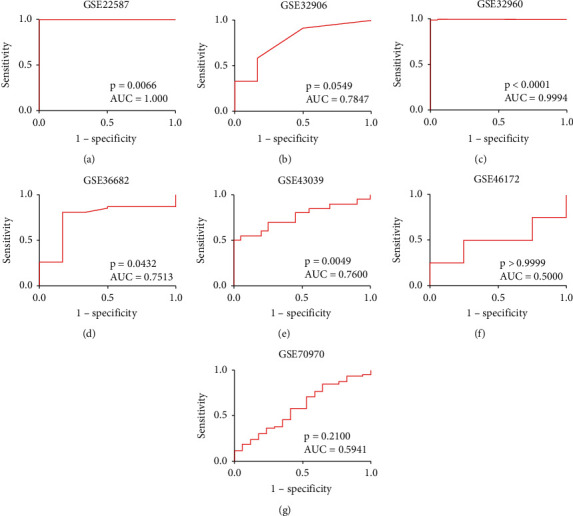
The receiver operating characteristic curves of miR-150-5p. The area under the curve of GSE22587, GSE32960, GSE36682, and GSE43039 suggested that miR-150-5p has a good ability to distinguish nasopharyngeal carcinoma tissues from non-tumor nasopharyngeal tissues.

**Figure 5:**
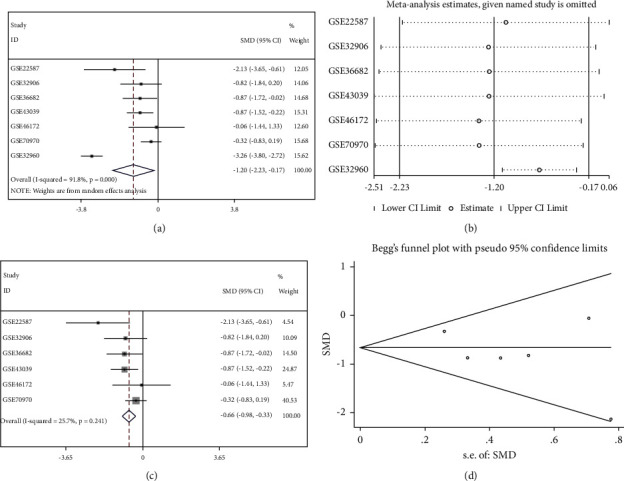
The expression of miR-150-5p is down-regulated in nasopharyngeal carcinoma. (a) Due to the large heterogeneity (*I*^2^ = 91.8%, *P* < 0.05), a random effect model is chosen for pooled analysis. The standardized mean difference of miR-150-5p is =−1.20. (b) The sensitivity analysis reveals that GSE32960 affected the stability of the standardized mean difference. (c) The heterogeneity is significantly reduced after excluding GSE32960 (*I*^2^ = 25.7%, *P*=0.241). And the standardized mean difference of miR-150-5p (a fixed effect model) is −0.66. (d) After excluding GSE32960, Begg's test shows no significant publication bias.

This study finally retrieved fourteen mRNA datasets and seven miRNA datasets from the GEO public database (Table 1). The selection process is visualized with Figures 1 and 2. The expression of miR-150-5p in individual datasets was represented using violin plots and ROC curves (Figures 3 and 4), and four datasets, including GSE22587, GSE32960, GSE36682, and GSE43039, showing that *P* < 0.05 in the violin plots and ROC curves. Since the individual studies were too small to draw stable conclusions, we integrated all theincluded datasets. Due to the large heterogeneity (*I*^2^ = 91.8%, *P* < 0.05) Figure 5(a), the random effect model was adopted. The SMD of miR-150-5p was =−1.20 (95% CI: −2.23, −0.17; Figure 5(a)). The sensitivity analysis revealed that GSE32960 largely affected the stability of the SMD (Figure 5(b)). The heterogeneity was significantly reduced after excluding GSE32960, and the fixed effect model was adopted (*I*^2^ = 25.7%, *P* = 0.241, Figure 5(c)). Therefore, the SMD was −0.66 (95% CI: −0.98, −0.33; Figure 5(c)). The above results revealed that the expression of miR-150-5p was distinctly down-regulated in NPC. Furthermore, Begg's test did not indicate obvious publication bias (Figure 5(d)).

**Figure 6:**
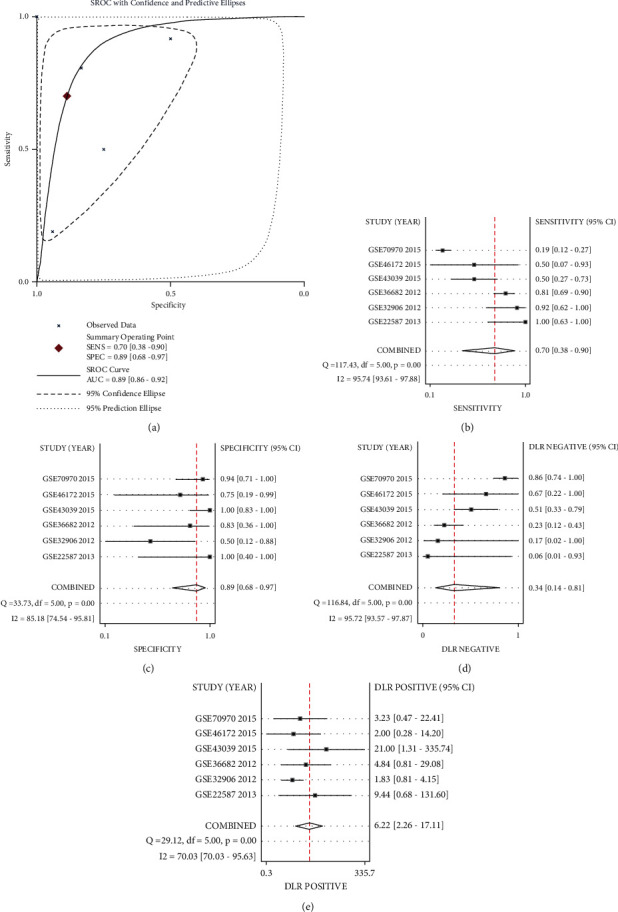
The summary receiver operating characteristic curve of miR-150-5p. The expression of miR-150-5p has excellent ability to distinguish nasopharyngeal carcinoma (NPC) tissue and non-NPC nasopharyngeal tissue. (a) SROC curve. (b) Pooled sensitivity. (c) Pooled specificity. (d) Negative diagnostic likelihood ratio. (e) Positive diagnostic likelihood ratio.

To further determine the ability of miR-150-5p to distinguish NPC and non-NPC nasopharyngeal tissues, we generated a sROC curve with the positive and negative diagnostic likelihood ratios, pooled sensitivity, and pooled specificity. The pooled AUC was 0.89 (95% CI; 0.86, 0.92; Figure 6(a)). The pooled sensitivity and pooled specificity were 0.70 (95% CI; 0.38, 0.90) and 0.89 (95% CI; 0.68, 0.97; Figures 6(b) and 6(c)), respectively. The negative and positive diagnostic likelihood ratios were 0.34 (95% CI; 0.14, 0.81) and 6.22 (95% CI; 2.26, 17.11; Figures 6(d) and 6(e)).

### 3.2. The Target Genes of miR-150-5p Were Obtained

**Figure 7:**
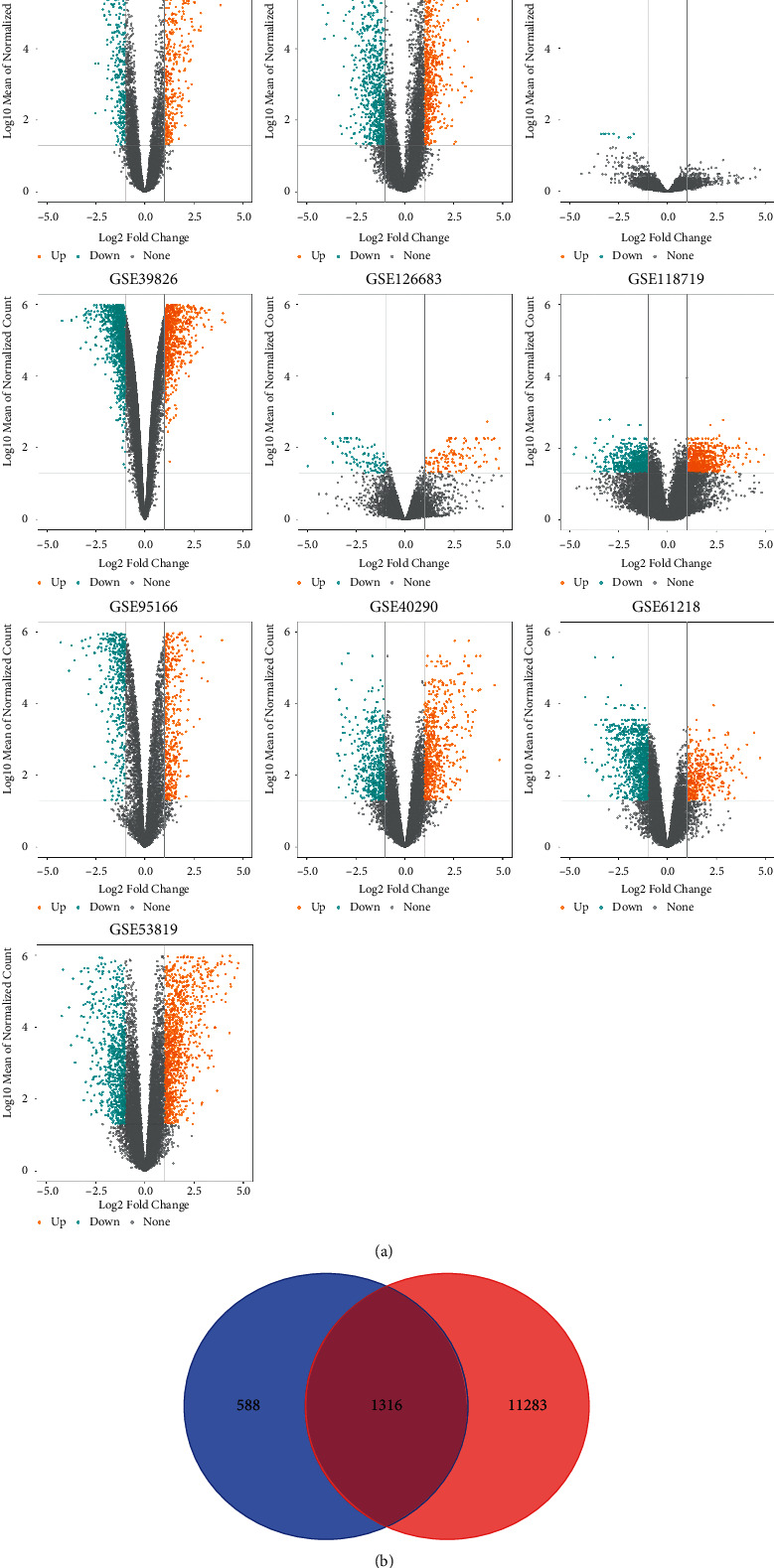
Identification of miR-150-5p target genes. (a) The volcano plots presents the DEGs. (b) 1316 target genes of miR-150-5p are obtained.

Overall, 12,599 predicted target genes were obtained from miRwalk. The DEGs of each datasets were visualized using volcano plots (Figure 7(a)). In addition, 1904 upregulated DEGs were screened out. The SMDs of 1904 up-regulated DEGs were displayed in Table S1. The genes that intersected two times were regarded as the target genes, and finally, 1316 target genes of miR-150-5p were obtained (Figure 7(b)).

### 3.3. The Results of Enrichment Analysis

**Figure 8:**
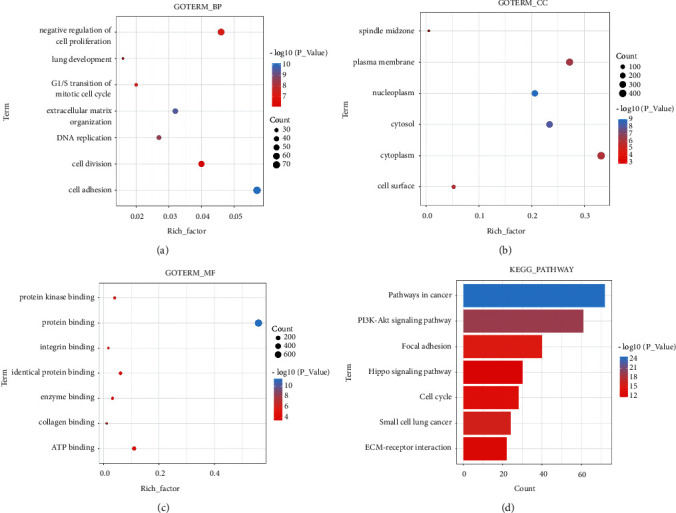
The GO and KEGG enrichment analysis of the target genes. (a) Statistically significant terms of biological process. (b) Statistically significant terms of cellular component. (c) Statistically significant terms of molecular function. (d) The significantly pathways of the Kyoto Encyclopedia of genes and genomes.

In this study, KEGG and GO analysis of 1316 target genes were performed using DAVID to further explore the molecular mechanisms of miR-150-5p in NPC. Regarding BP, the target genes mainly focused on G1/S transition of mitotic cell cycle, cell division, and DNA replication (Figure 8(a)). For the CC, the target genes focused on the nucleoplasm, cytoplasm, and spindle midzone (Figure 8(b)). Regarding MF, the target genes focused on the protein binding and protein-kinase binding (Figure 8(c)). As for KEGG, a total of seven important pathways were identified, including “pathways in cancer” (Figure 8(d)).

### 3.4. The Hub Genes of the Network

**Figure 9:**
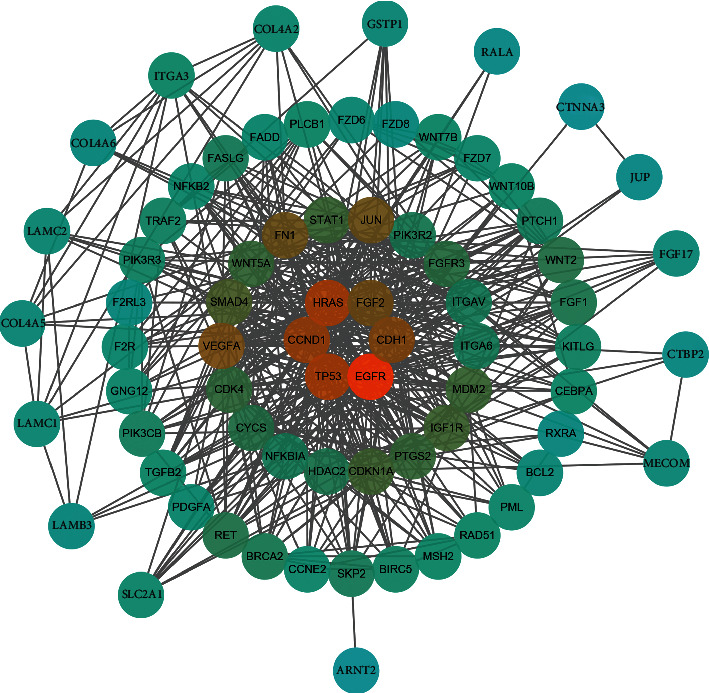
The PPI network is constructed based on the 72 target genes of “pathways in cancer”. Six hub genes, including EGFR, TP53, HRAS, CCND1, CDH1, and FGF2, are selected using Centiscape plug-in.

The PPI network was constructed using the 72 target genes of “pathways in cancer” (Figure 9). The Centiscape were adopted to screen hub genes. Six hub genes were screened, including EGFR, TP53, HRAS, CCND1, CDH1, and FGF2.

### 3.5. The mRNA Expression of the Six Hub Genes

**Figure 10:**
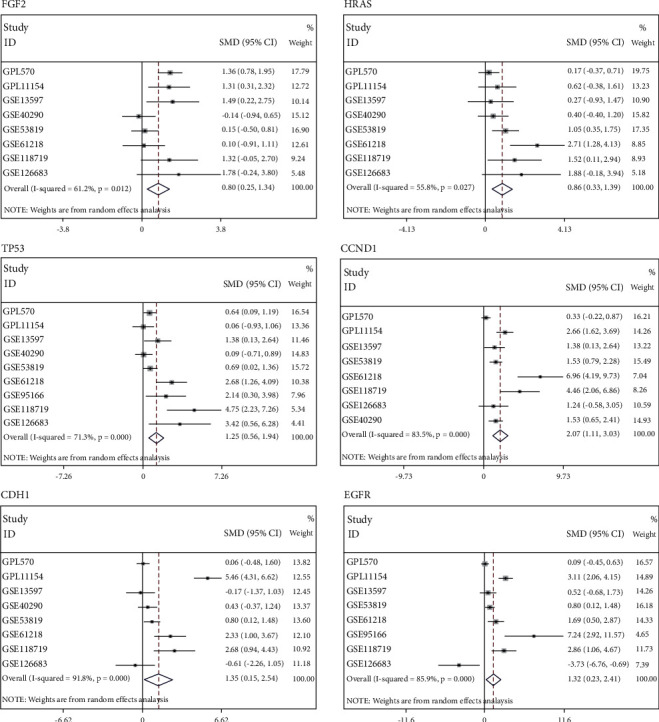
The expression of all six hub genes is significantly up-regulated in nasopharyngeal carcinoma.

The mRNA expression of the six hub genes, including EGFR, TP53, HRAS, CCND1, CDH1, and FGF2, was assessed using SMD. As shown in Figure 10, SMDs of all six hub genes exceeded 0, and 95% CI was over 0. The above results indicated that the six genes were highly expressed in NPC.

## 4. Discussion

This study comprehensively explored the role of miR-150-5p in NPC and confirmed that the down-regulation of miR-150-5p promoted the tumorigenesis of NPC by regulating the target genes.

NPC is a malignant tumor prevalent in Southeast Asia and Southern China [18]. Due to the lack of biomarkers for early diagnosis and a high degree of malignancy, most patients have local metastases at initial diagnosis [19, 20]. Therefore, exploring the pathogenesis and discovering new biomarkers are imperative. miRNA is a type of endogenous RNA with 17–23 nucleotides in length [13]. It regulates the transcription of target genes by combining with the 3′-UTR region, thereby modulating cell growth, differentiation, and apoptosis [21]. Many miRNAs are aberrantly expressed in NPC and regulate relevant target genes, thus affecting proliferation, invasion, and metastasis of tumor cells. For example, miR-142 is highly expressed in NPC and enhances the invasiveness and proliferation of NPC by inhibiting PTEN expression [22]. MiR-129-5p inhibits NPC lymph node metastasis and lymphangiogenesis by reducing ZIC2 expression [23]. MiR-18a plays its carcinogenic role by inhibiting SMG1 expression and activating the mTOR pathway in NPC [24]. LINC01551 promotes the metastatic ability of NPC by regulating miR-132-5p [25]. These indicate that miRNAs are critical in NPC tumorigenesis and development. Exploring the mechanism of miRNAs contributes to the treatment and diagnosis of NPC.

The miR-150-5p has been demonstrated to have an important effect on proliferation, invasion, and metastasis of multiple tumors. For example, miR-150-5p contributes to the epithelial–mesenchymal transition and cell growth of cervical carcinoma cells by regulating SRCIN1 [17]. In addition, miR-150-5p enhances the metastasis and proliferation of gastric cancer by interacting with circLMTK2 [26]. However, the mechanisms of miR-150-5p in NPC remain unclear. This study is helpful in revealing the mechanism of miR-150-5p in NPC tumorigenesis and progression.

In this study, a pooled analysis based on 539 NPC samples and 75 non-NPC nasopharyngeal samples demonstrated that the expression of miR-150-5p was down-regulated in NPC. The sROC curve indicated that miR-150-5p had high specificity for differentiating NPC from non-NPC nasopharyngeal samples. Subsequently, 1316 target genes were screened to perform GO and KEGG enrichment analysis. Finally, 72 target genes of “pathways in cancer” were selected to construct the PPI network, and six hub genes were screened, including EGFR, TP53, HRAS, CCND1, CDH1, and FGF2.

EGFR was demonstrated to have an important effect on the proliferation, invasion, and metastasis of multiple tumors [27]. After being activated by ligands, EGFR dimerizes and triggers the production of tyrosine kinase activity to activate downstream signal pathways and participate in cell proliferation, differentiation, division, survival, and cancer development [28, 29]. Leong et al. found that EGFR was overexpressed in most NPC patients [30]. Nimotuzumab is a monoclonal antibody against EGFR. Combined treatment with nimotuzumab and celecoxib can enhance the radiosensitivity of CNE2 cells by inhibiting the EGFR pathway [31]. A retrospective analysis showed that nimotuzumab combined with concurrent chemoradiotherapy achieved exciting efficacy in the treatment of locally advanced NPC [32]. TP53 is a tumor suppressor gene that encodes a protein with a molecular weight of 53 kDa. It participates in the processes of cell differentiation, proliferation, apoptosis, senescence, autophagy, inflammation, and metabolism by regulating the transcription of various genes [33]. TP53 is abnormally expressed in thyroid carcinoma, gastric cancer, and esophageal carcinoma and is associated with tumorigenesis and progression [34–36]. CCND1 is an important regulator of cell cycle [37, 38]. MiR-584 targets CCND1 to inhibit invasion and proliferation of pancreatic carcinoma [39]. In colorectal carcinoma, miR-628-5p can downregulate CCND1 expression to induce apoptosis and restrain cell proliferation [40]. In ovarian cancer, the inhibition of CCND1 expression by cisplatin can reduce cell proliferation and increase cell apoptosis [41]. LncRNA HCG18 can act as a ceRNA of miR-140 to upregulate the expression of CCND1 and promote the progression of NPC [42]. HRAS is an important member of the RAS oncogene family. As an essential part of the signal network that controls cell proliferation, differentiation, and survival, the RAS protein plays a vital role in tumorigenesis and progression [43]. In NPC, circZNF609 affects cell proliferation, invasion, migration, and glycolysis by regulating the miR-338-3p/HRAS axis [44]. CDH1 encodes E-cadherin. As a calcium-dependent transmembrane protein, E-cadherin is involved in cell–cell adhesion and has an important effect in maintaining epithelial structure and function [45]. As a vital angiogenic factor, FGF2 induces the migration and growth of endothelial cells [46]. FGF2 is produced by tumor cells and surrounding stromal cells, and promotes angiogenesis, migration, proliferation, and invasion of tumor cells [47].

All in all, the expression of miR-150-5p was down-regulated in NPC and affected tumorigenesis and progression of NPC by regulating target genes. We will further validate the effects of miR-150-5p on the six hub genes by experiments and further explore the key pathways.

## 5. Conclusions

This study confirmed the low expression of miR-150-5p in NPC, and that miR-150-5p regulated the tumorigenesis and progression of NPC by targeting EGFR, TP53, HRAS, CCND1, CDH1 and FGF2.

## Figures and Tables

**Table 1:** Characteristics of GEO datasets included in this study.

Publication year	ID of datasets	Platform	Cancer group	Normal controls
2013	GSE22587	GPL8933	8	4
2012	GSE32906	GPL11350	12	6
2012	GSE32960	GPL14722	312	18
2012	GSE36682	GPL15311	62	6
2015	GSE43039	GPL16414	20	20
2014	GSE46172	GPL16770	4	4
2015	GSE70970	GPL20699	121	17
2009	GSE13597	GPL96	25	3
2016	GSE40290	GPL8380	25	8
2014	GSE53819	GPL6480	18	18
2020	GSE61218	GPL19061	10	6
2019	GSE95166	GPL15314	4	4
2019	GSE126683	GPL16956	3	3
2019	GSE118719	GPL20301	7	4
2013	GSE39826	GPL6244	3	3
2016	GSE68799	GPL11154	42	4
2014	GSE63381	GPL11154	4	0
2017	GSE102349	GPL11154	113	0
2017	GSE64634	GPL570	12	4
2012	GSE34573	GPL570	15	3
2008	GSE12452	GPL570	31	10

## Data Availability

All datasets were obtained from GEO database. The corresponding author can be contacted to obtain relevant data used in this study.

## Supplementary Materials

Supplementary Materials Table S1. The standardized mean differences of 1904 up-regulated differentially expressed genes.
