# Supplementary material for: Downregulated miR-150-5p in the Tissue of Nasopharyngeal Carcinoma
Source: Genet Res (Camb). 2022 Sep 5;2022:2485055. doi: 10.1155/2022/2485055 (PMC9467814; doi:10.1155/2022/2485055)
Supplement: Supplementary Materials — Table S1. The standardized mean differences of 1904 up-regulated differentially expressed genes. [file 2485055.f1.docx]

**Table S1**. The standardized mean differences of 1904 up-regulated differentially expressed genes.

| **Gene symbol** | **SMD** | **95% confidence interval** | **P** |
| --- | --- | --- | --- |
| AACSP1 | 1.0005 | [0.5368;1.4642] | <0.0001 |
| ABCA12 | 0.7706 | [0.4550;1.0862] | <0.0001 |
| ABCA17P | 1.1018 | [0.1902;2.0133] | 0.0178 |
| ABCA3 | 1.1588 | [0.8374;1.4802] | <0.0001 |
| ABCB6 | 0.7424 | [0.4251;1.0597] | <0.0001 |
| ABCC1 | 1.2654 | [0.4952;2.0355] | 0.0013 |
| ABCC4 | 0.9857 | [0.6665;1.3049] | <0.0001 |
| ABCC5 | 0.8448 | [0.5306;1.1589] | <0.0001 |
| ABHD11 | 0.7381 | [0.0750;1.4011] | 0.0291 |
| ABLIM2 | 0.6905 | [0.3701;1.0109] | <0.0001 |
| ABRACL | 1.3094 | [0.7908;1.8281] | <0.0001 |
| ABTB2 | 0.9725 | [0.6514;1.2936] | <0.0001 |
| ACACA | 1.2051 | [0.4922;1.9181] | 0.0009 |
| ACADSB | 0.3381 | [0.0350;0.6412] | 0.0288 |
| ACER3 | 0.9438 | [0.5826;1.3049] | <0.0001 |
| ACOXL | 0.6132 | [0.0440;1.1824] | 0.0347 |
| ACSL4 | 0.3718 | [0.0647;0.6788] | 0.0176 |
| ACTR3B | 0.8643 | [0.5281;1.2005] | <0.0001 |
| ACVR1B | 1.2425 | [0.6863;1.7986] | <0.0001 |
| ADA | 0.7593 | [0.2523;1.2664] | 0.0033 |
| ADAM12 | 0.5649 | [0.0557;1.0741] | 0.0297 |
| ADAM18 | 0.3924 | [0.0599;0.7249] | 0.0207 |
| ADAM22 | 1.2309 | [0.5861;1.8756] | 0.0002 |
| ADAM23 | 1.1743 | [0.6339;1.7146] | <0.0001 |
| ADAM9 | 0.9259 | [0.1807;1.6712] | 0.0149 |
| ADAMDEC1 | 0.5568 | [0.0676;1.0461] | 0.0257 |
| ADAMTS1 | 0.3474 | [0.0405;0.6544] | 0.0265 |
| ADAMTS17 | 0.8767 | [0.5251;1.2283] | <0.0001 |
| ADAMTS2 | 0.5596 | [0.0521;1.0671] | 0.0307 |
| ADAMTSL4 | 0.3818 | [0.0495;0.7141] | 0.0243 |
| ADCY10 | 0.8102 | [0.4693;1.1511] | <0.0001 |
| ADK | 0.5917 | [0.2826;0.9008] | 0.0002 |
| ADNP2 | 1.4029 | [1.0334;1.7724] | <0.0001 |
| ADORA3 | 0.6015 | [0.2851;0.9180] | 0.0002 |
| ADTRP | 0.8278 | [0.3696;1.2859] | 0.0004 |
| AEBP2 | 1.2105 | [0.5659;1.8550] | 0.0002 |
| AFAP1 | 0.9674 | [0.1635;1.7712] | 0.0183 |
| AFAP1-AS1 | 0.9173 | [0.5192;1.3155] | <0.0001 |
| AFAP1L2 | 0.3963 | [0.0515;0.7411] | 0.0243 |
| AFMID | 1.1634 | [0.1602;2.1667] | 0.023 |
| AGAP1 | 1.2875 | [0.4945;2.0805] | 0.0015 |
| AGPAT5 | 0.8872 | [0.5428;1.2317] | <0.0001 |
| AGTPBP1 | 0.4682 | [0.1615;0.7749] | 0.0028 |
| AHCTF1 | 1.3322 | [0.9776;1.6868] | <0.0001 |
| AHCY | 1.5186 | [1.1847;1.8525] | <0.0001 |
| AIM1 | 0.4743 | [0.1383;0.8103] | 0.0057 |
| AIMP2 | 1.4209 | [0.6379;2.2038] | 0.0004 |
| AJUBA | 1.4571 | [0.6555;2.2586] | 0.0004 |
| AKAP1 | 0.6917 | [0.3760;1.0074] | <0.0001 |
| AKIRIN2 | 0.6698 | [0.3178;1.0218] | 0.0002 |
| AKT1S1 | 1.1876 | [0.3311;2.0442] | 0.0066 |
| ALDH1L2 | 0.4714 | [0.1277;0.8150] | 0.0072 |
| ALDH2 | 0.6429 | [0.0277;1.2581] | 0.0406 |
| ALK | 0.4331 | [0.1256;0.7407] | 0.0058 |
| ALOX12B | 0.392 | [0.0810;0.7029] | 0.0135 |
| AMH | 1.0834 | [0.6707;1.4961] | <0.0001 |
| AMOTL1 | 1.0786 | [0.3953;1.7620] | 0.002 |
| AMTN | 0.9031 | [0.3000;1.5063] | 0.0033 |
| AMZ2P1 | 0.4712 | [0.1028;0.8395] | 0.0122 |
| ANAPC7 | 1.0309 | [0.2950;1.7669] | 0.006 |
| ANGPT2 | 0.9022 | [0.5917;1.2126] | <0.0001 |
| ANGPTL4 | 0.4135 | [0.1063;0.7206] | 0.0083 |
| ANKH | 0.867 | [0.5516;1.1824] | <0.0001 |
| ANKLE2 | 1.1479 | [0.7910;1.5047] | <0.0001 |
| ANKRD13B | 0.7391 | [0.3839;1.0944] | <0.0001 |
| ANKRD20A8P | 1.0049 | [0.3869;1.6230] | 0.0014 |
| ANKRD22 | 2.1925 | [1.0259;3.3592] | 0.0002 |
| ANKRD32 | 0.8106 | [0.1252;1.4961] | 0.0205 |
| ANKRD33B | 0.9376 | [0.5809;1.2943] | <0.0001 |
| ANKRD50 | 0.9356 | [0.2580;1.6132] | 0.0068 |
| ANKRD6 | 0.5655 | [0.2552;0.8757] | 0.0004 |
| ANLN | 1.5383 | [1.1785;1.8981] | <0.0001 |
| ANO1 | 0.6102 | [0.2735;0.9468] | 0.0004 |
| ANTXR1 | 0.5998 | [0.2918;0.9078] | 0.0001 |
| ANXA4 | 1.7957 | [0.9062;2.6851] | <0.0001 |
| AP3B2 | 0.867 | [0.1543;1.5797] | 0.0171 |
| AP3M2 | 1.0653 | [0.7409;1.3897] | <0.0001 |
| APOBEC3B | 0.7824 | [0.4359;1.1289] | <0.0001 |
| APOBEC3H | 1.334 | [0.4331;2.2349] | 0.0037 |
| APOC1 | 0.8466 | [0.0606;1.6326] | 0.0348 |
| APOL1 | 0.4627 | [0.1288;0.7966] | 0.0066 |
| APOL6 | 0.7872 | [0.1736;1.4007] | 0.0119 |
| APP | 1.0438 | [0.1718;1.9158] | 0.019 |
| AQP9 | 0.667 | [0.3586;0.9754] | <0.0001 |
| ARHGAP10 | 0.8063 | [0.4950;1.1177] | <0.0001 |
| ARHGAP11A | 0.8016 | [0.1815;1.4218] | 0.0113 |
| ARHGAP26 | 0.7749 | [0.4603;1.0895] | <0.0001 |
| ARHGAP28 | 0.77 | [0.4356;1.1044] | <0.0001 |
| ARHGEF19 | 1.3444 | [0.4885;2.2003] | 0.0021 |
| ARHGEF35 | 0.611 | [0.1748;1.0473] | 0.006 |
| ARID2 | 0.9827 | [0.6241;1.3413] | <0.0001 |
| ARL14 | 1.0377 | [0.6968;1.3786] | <0.0001 |
| ARL4A | 0.6544 | [0.0704;1.2384] | 0.0281 |
| ARL5B | 0.9986 | [0.3121;1.6851] | 0.0044 |
| ARL6IP6 | 1.2153 | [0.8426;1.5880] | <0.0001 |
| ARNT2 | 1.5378 | [0.9737;2.1020] | <0.0001 |
| ARNTL2 | 1.1526 | [0.8081;1.4972] | <0.0001 |
| ASB9 | 1.5129 | [0.9476;2.0783] | <0.0001 |
| ASCL4 | 1.1352 | [0.5345;1.7359] | 0.0002 |
| ASF1B | 0.6937 | [0.3791;1.0083] | <0.0001 |
| ASNS | 0.8159 | [0.5001;1.1318] | <0.0001 |
| ASPM | 1.6154 | [0.9071;2.3236] | <0.0001 |
| ASPN | 0.8446 | [0.5326;1.1565] | <0.0001 |
| ATAD2 | 1.6785 | [0.8514;2.5057] | <0.0001 |
| ATAD5 | 0.9777 | [0.1895;1.7660] | 0.0151 |
| ATF3 | 0.771 | [0.0412;1.5009] | 0.0384 |
| ATF5 | 1.2037 | [0.5795;1.8280] | 0.0002 |
| ATIC | 0.9453 | [0.2559;1.6348] | 0.0072 |
| ATL2 | 1.4006 | [0.5955;2.2057] | 0.0007 |
| ATN1 | 0.8842 | [0.2401;1.5283] | 0.0071 |
| ATP11C | 1.3254 | [0.9515;1.6994] | <0.0001 |
| ATP13A3 | 0.4324 | [0.0990;0.7658] | 0.011 |
| ATP1B1 | 1.1529 | [0.0204;2.2854] | 0.046 |
| ATP1B3 | 0.5755 | [0.2652;0.8857] | 0.0003 |
| ATP2B4 | 0.7344 | [0.1136;1.3553] | 0.0204 |
| ATP2C1 | 2.0974 | [1.2529;2.9419] | <0.0001 |
| ATP6V0A2 | 0.4469 | [0.1273;0.7666] | 0.0061 |
| ATP6V1B2 | 0.9254 | [0.6084;1.2424] | <0.0001 |
| ATP6V1C2 | 0.379 | [0.0614;0.6965] | 0.0193 |
| ATP8B4 | 0.8078 | [0.4967;1.1189] | <0.0001 |
| AUNIP | 1.34 | [0.1786;2.5015] | 0.0237 |
| AURKA | 1.4717 | [0.5610;2.3824] | 0.0015 |
| AURKB | 0.962 | [0.2828;1.6411] | 0.0055 |
| AXL | 0.7765 | [0.4649;1.0881] | <0.0001 |
| B3GALNT2 | 1.3033 | [0.6790;1.9275] | <0.0001 |
| B4GALNT4 | 1.3885 | [0.5332;2.2438] | 0.0015 |
| B4GALT2 | 0.6244 | [0.3104;0.9383] | <0.0001 |
| B4GALT5 | 1.0798 | [0.2703;1.8893] | 0.0089 |
| B4GALT6 | 1.1499 | [0.8259;1.4738] | <0.0001 |
| BAHD1 | 0.7575 | [0.4461;1.0690] | <0.0001 |
| BAI2 | 0.7528 | [0.1993;1.3064] | 0.0077 |
| BAMBI | 0.3418 | [0.0380;0.6456] | 0.0275 |
| BCHE | 0.5794 | [0.2711;0.8877] | 0.0002 |
| BCL2 | 0.498 | [0.1911;0.8049] | 0.0015 |
| BCL2L11 | 0.9949 | [0.6715;1.3184] | <0.0001 |
| BCL2L14 | 0.5811 | [0.0642;1.0980] | 0.0276 |
| BCL7A | 0.5418 | [0.2104;0.8733] | 0.0014 |
| BDNF | 0.7084 | [0.3762;1.0407] | <0.0001 |
| BEND3 | 0.9848 | [0.3232;1.6465] | 0.0035 |
| BID | 0.7872 | [0.4767;1.0977] | <0.0001 |
| BIRC5 | 1.035 | [0.0473;2.0228] | 0.04 |
| BLMH | 0.633 | [0.3235;0.9424] | <0.0001 |
| BLVRA | 1.0509 | [0.7047;1.3971] | <0.0001 |
| BMP7 | 0.6051 | [0.2963;0.9138] | 0.0001 |
| BMPR2 | 0.5375 | [0.2033;0.8718] | 0.0016 |
| BOD1 | 0.95 | [0.5927;1.3073] | <0.0001 |
| BOLA3 | 0.8351 | [0.4861;1.1841] | <0.0001 |
| BORA | 0.7183 | [0.3544;1.0822] | 0.0001 |
| BRCA1 | 1.5464 | [0.7937;2.2990] | <0.0001 |
| BRCA2 | 1.0471 | [0.4342;1.6601] | 0.0008 |
| BRD8 | 0.4239 | [0.1161;0.7316] | 0.0069 |
| BRF2 | 0.4427 | [0.1359;0.7495] | 0.0047 |
| BRI3BP | 1.1854 | [0.1764;2.1944] | 0.0213 |
| BRIP1 | 1.5759 | [1.2255;1.9263] | <0.0001 |
| BSG | 0.8664 | [0.0981;1.6347] | 0.0271 |
| BST2 | 0.8547 | [0.5394;1.1700] | <0.0001 |
| BTBD3 | 1.1655 | [0.4535;1.8775] | 0.0013 |
| BTG3 | 1.7921 | [1.0495;2.5348] | <0.0001 |
| BUB1 | 1.1555 | [0.4905;1.8206] | 0.0007 |
| BUB1B | 1.451 | [0.6558;2.2461] | 0.0003 |
| BZW2 | 0.7695 | [0.1653;1.3737] | 0.0126 |
| C10orf10 | 0.7231 | [0.3834;1.0627] | <0.0001 |
| C11orf96 | 0.4266 | [0.0866;0.7665] | 0.0139 |
| C12orf5 | 1.1587 | [0.5564;1.7610] | 0.0002 |
| C12orf54 | 0.7669 | [0.0708;1.4629] | 0.0308 |
| C12orf73 | 1.2323 | [0.5399;1.9246] | 0.0005 |
| C14orf132 | 0.9587 | [0.6428;1.2746] | <0.0001 |
| C15orf54 | 0.58 | [0.1331;1.0269] | 0.011 |
| C17orf53 | 0.666 | [0.0252;1.3069] | 0.0417 |
| C17orf75 | 0.8802 | [0.2201;1.5404] | 0.009 |
| C19orf48 | 1.0089 | [0.2342;1.7836] | 0.0107 |
| C1orf109 | 0.6697 | [0.3366;1.0027] | <0.0001 |
| C1orf112 | 1.0028 | [0.6605;1.3451] | <0.0001 |
| C1orf131 | 0.4478 | [0.1012;0.7945] | 0.0113 |
| C1orf53 | 0.9177 | [0.2517;1.5837] | 0.0069 |
| C1QB | 0.9018 | [0.3247;1.4788] | 0.0022 |
| C1QBP | 1.0102 | [0.6829;1.3374] | <0.0001 |
| C1QC | 0.8468 | [0.4922;1.2014] | <0.0001 |
| C1QTNF1 | 0.6293 | [0.3195;0.9392] | <0.0001 |
| C20orf24 | 0.9411 | [0.2121;1.6701] | 0.0114 |
| C2orf69 | 0.7479 | [0.3964;1.0995] | <0.0001 |
| C3orf14 | 0.6141 | [0.3036;0.9246] | 0.0001 |
| C3orf58 | 0.8484 | [0.4805;1.2163] | <0.0001 |
| C3orf80 | 1.2483 | [0.3455;2.1512] | 0.0067 |
| C4orf46 | 1.2107 | [0.3079;2.1134] | 0.0086 |
| C5 | 0.5296 | [0.2209;0.8383] | 0.0008 |
| C5AR1 | 0.4738 | [0.1417;0.8059] | 0.0052 |
| C5orf30 | 0.8008 | [0.4450;1.1565] | <0.0001 |
| C5orf34 | 0.9426 | [0.2582;1.6269] | 0.0069 |
| C6orf141 | 2.04 | [1.6368;2.4431] | <0.0001 |
| C8orf4 | 1.0666 | [0.5043;1.6288] | 0.0002 |
| C9orf3 | 0.7888 | [0.1944;1.3833] | 0.0093 |
| CA2 | 0.3185 | [0.0135;0.6236] | 0.0407 |
| CA9 | 0.7572 | [0.1731;1.3414] | 0.0111 |
| CABLES1 | 0.7948 | [0.1791;1.4105] | 0.0114 |
| CACNA2D1 | 1.0176 | [0.3540;1.6813] | 0.0027 |
| CAD | 1.0291 | [0.3316;1.7267] | 0.0038 |
| CALD1 | 0.7144 | [0.1888;1.2400] | 0.0077 |
| CALN1 | 0.9435 | [0.5888;1.2982] | <0.0001 |
| CALU | 1.036 | [0.7124;1.3597] | <0.0001 |
| CAMK1G | 0.73 | [0.2114;1.2486] | 0.0058 |
| CAMKK1 | 0.7885 | [0.2031;1.3739] | 0.0083 |
| CAMSAP2 | 1.3229 | [0.5019;2.1438] | 0.0016 |
| CAMTA1 | 0.9499 | [0.6328;1.2670] | <0.0001 |
| CARD18 | 0.674 | [0.2937;1.0543] | 0.0005 |
| CASK | 1.2229 | [0.8968;1.5490] | <0.0001 |
| CAV1 | 0.6478 | [0.0694;1.2262] | 0.0282 |
| CAV2 | 0.9994 | [0.4246;1.5743] | 0.0007 |
| CBS | 0.583 | [0.2719;0.8941] | 0.0002 |
| CBX3 | 1.0736 | [0.7513;1.3958] | <0.0001 |
| CBX8 | 0.8473 | [0.0759;1.6187] | 0.0313 |
| CCDC138 | 0.7474 | [0.0251;1.4697] | 0.0425 |
| CCDC14 | 0.9092 | [0.1877;1.6306] | 0.0135 |
| CCDC150 | 1.3482 | [0.4274;2.2690] | 0.0041 |
| CCDC25 | 0.409 | [0.0750;0.7431] | 0.0164 |
| CCDC28B | 1.0854 | [0.7398;1.4311] | <0.0001 |
| CCDC34 | 1.3934 | [0.5720;2.2149] | 0.0009 |
| CCDC58 | 1.0396 | [0.4085;1.6707] | 0.0012 |
| CCDC77 | 0.7919 | [0.1954;1.3885] | 0.0093 |
| CCDC8 | 0.4104 | [0.0956;0.7252] | 0.0106 |
| CCL11 | 0.5842 | [0.2746;0.8939] | 0.0002 |
| CCL2 | 0.7259 | [0.4177;1.0341] | <0.0001 |
| CCL20 | 1.2108 | [0.4938;1.9277] | 0.0009 |
| CCL3 | 0.6342 | [0.1859;1.0826] | 0.0056 |
| CCL4 | 0.8363 | [0.2438;1.4288] | 0.0057 |
| CCL7 | 0.8635 | [0.5488;1.1782] | <0.0001 |
| CCL8 | 1.2904 | [0.9648;1.6159] | <0.0001 |
| CCNA2 | 1.2405 | [0.5189;1.9620] | 0.0008 |
| CCNB1 | 1.021 | [0.3351;1.7068] | 0.0035 |
| CCNB2 | 1.6957 | [0.8462;2.5451] | <0.0001 |
| CCND1 | 1.9099 | [0.9603;2.8595] | <0.0001 |
| CCND2 | 1.0408 | [0.7211;1.3606] | <0.0001 |
| CCNE2 | 1.6012 | [0.9799;2.2225] | <0.0001 |
| CCNF | 1.1394 | [0.5393;1.7395] | 0.0002 |
| CCNJL | 1.1518 | [0.4425;1.8611] | 0.0015 |
| CCNYL2 | 0.6347 | [0.1288;1.1406] | 0.0139 |
| CCR1 | 0.9594 | [0.6413;1.2775] | <0.0001 |
| CCR8 | 1.1007 | [0.2339;1.9675] | 0.0128 |
| CCT2 | 1.0971 | [0.7691;1.4252] | <0.0001 |
| CCT3 | 0.9219 | [0.5100;1.3338] | <0.0001 |
| CCT5 | 0.8128 | [0.2819;1.3437] | 0.0027 |
| CCT6A | 1.6767 | [1.0471;2.3063] | <0.0001 |
| CD109 | 0.8749 | [0.5202;1.2295] | <0.0001 |
| CD163 | 0.6682 | [0.3396;0.9968] | <0.0001 |
| CD1A | 0.687 | [0.3830;0.9909] | <0.0001 |
| CD200 | 0.9151 | [0.6005;1.2297] | <0.0001 |
| CD274 | 1.193 | [0.5450;1.8410] | 0.0003 |
| CD276 | 1.1825 | [0.4672;1.8978] | 0.0012 |
| CD2AP | 0.3203 | [0.0156;0.6250] | 0.0393 |
| CD70 | 1.2184 | [0.5233;1.9135] | 0.0006 |
| CD9 | 0.9936 | [0.1477;1.8395] | 0.0213 |
| CDC25A | 0.8387 | [0.2100;1.4675] | 0.0089 |
| CDC25C | 1.0113 | [0.4329;1.5897] | 0.0006 |
| CDC42BPA | 0.7865 | [0.2681;1.3048] | 0.0029 |
| CDC42BPG | 0.819 | [0.2014;1.4366] | 0.0094 |
| CDC42EP2 | 0.5406 | [0.0004;1.0808] | 0.0498 |
| CDC42EP4 | 0.9815 | [0.3466;1.6164] | 0.0024 |
| CDC45 | 1.2492 | [0.5619;1.9364] | 0.0004 |
| CDC6 | 1.4424 | [0.8583;2.0266] | <0.0001 |
| CDC7 | 0.8598 | [0.2772;1.4425] | 0.0038 |
| CDCA2 | 1.0731 | [0.3571;1.7891] | 0.0033 |
| CDCA4 | 0.7199 | [0.0842;1.3555] | 0.0264 |
| CDCA5 | 0.9425 | [0.1083;1.7767] | 0.0268 |
| CDCA7 | 1.1778 | [0.3346;2.0210] | 0.0062 |
| CDH1 | 1.2678 | [0.0929;2.4426] | 0.0344 |
| CDH13 | 0.374 | [0.0665;0.6815] | 0.0171 |
| CDH23 | 0.9704 | [0.2804;1.6605] | 0.0058 |
| CDK1 | 1.831 | [1.1640;2.4979] | <0.0001 |
| CDK16 | 0.8451 | [0.1006;1.5896] | 0.0261 |
| CDK18 | 1.6609 | [0.8820;2.4398] | <0.0001 |
| CDK2AP1 | 1.3326 | [1.0042;1.6609] | <0.0001 |
| CDK4 | 1.4199 | [0.7066;2.1333] | <0.0001 |
| CDKN1A | 1.0285 | [0.4554;1.6016] | 0.0004 |
| CDKN2AIPNL | 0.6704 | [0.0989;1.2419] | 0.0215 |
| CDON | 0.8288 | [0.5142;1.1434] | <0.0001 |
| CDT1 | 1.1953 | [0.2667;2.1239] | 0.0116 |
| CEACAM20 | 1.1203 | [0.6466;1.5940] | <0.0001 |
| CEBPA | 0.7835 | [0.2389;1.3281] | 0.0048 |
| CEL | 0.9592 | [0.3362;1.5823] | 0.0025 |
| CELSR2 | 0.8938 | [0.0914;1.6963] | 0.029 |
| CENPA | 1.1842 | [0.3245;2.0438] | 0.0069 |
| CENPE | 1.1079 | [0.3582;1.8575] | 0.0038 |
| CENPF | 1.6535 | [1.0080;2.2990] | <0.0001 |
| CENPH | 1.3105 | [0.9611;1.6600] | <0.0001 |
| CENPI | 1.3008 | [0.4724;2.1292] | 0.0021 |
| CENPJ | 1.0744 | [0.3574;1.7915] | 0.0033 |
| CENPK | 0.7652 | [0.4202;1.1102] | <0.0001 |
| CENPL | 1.254 | [0.3915;2.1166] | 0.0044 |
| CENPN | 1.3654 | [0.6250;2.1057] | 0.0003 |
| CENPO | 0.9677 | [0.2275;1.7079] | 0.0104 |
| CENPU | 1.8686 | [1.3155;2.4217] | <0.0001 |
| CENPW | 1.5192 | [0.8391;2.1993] | <0.0001 |
| CEP135 | 1.1229 | [0.7685;1.4772] | <0.0001 |
| CEP152 | 1.0933 | [0.2050;1.9816] | 0.0159 |
| CEP55 | 1.4212 | [0.5901;2.2523] | 0.0008 |
| CEP70 | 1.2216 | [0.8745;1.5688] | <0.0001 |
| CEP78 | 0.7434 | [0.3891;1.0977] | <0.0001 |
| CEP85 | 0.3492 | [0.0013;0.6972] | 0.0492 |
| CERS6 | 1.1769 | [0.7955;1.5583] | <0.0001 |
| CFHR4 | 0.3857 | [0.0247;0.7468] | 0.0363 |
| CHAC1 | 0.7221 | [0.3879;1.0562] | <0.0001 |
| CHAC2 | 0.7447 | [0.3952;1.0941] | <0.0001 |
| CHAF1A | 1.0047 | [0.3444;1.6650] | 0.0029 |
| CHAF1B | 1.7095 | [1.0796;2.3393] | <0.0001 |
| CHEK1 | 0.895 | [0.1300;1.6599] | 0.0218 |
| CHEK2 | 1.0949 | [0.5169;1.6729] | 0.0002 |
| CHI3L1 | 0.8229 | [0.3198;1.3260] | 0.0013 |
| CHML | 0.3782 | [0.0737;0.6827] | 0.0149 |
| CHN1 | 0.5296 | [0.2208;0.8384] | 0.0008 |
| CHRNA5 | 1.0947 | [0.4527;1.7367] | 0.0008 |
| CHRNA6 | 0.7889 | [0.0998;1.4780] | 0.0248 |
| CHST3 | 1.0158 | [0.6975;1.3342] | <0.0001 |
| CHSY3 | 0.6476 | [0.3025;0.9927] | 0.0002 |
| CHTF18 | 0.8959 | [0.1917;1.6001] | 0.0127 |
| CISD2 | 0.8926 | [0.5317;1.2534] | <0.0001 |
| CIT | 1.6637 | [0.6604;2.6670] | 0.0012 |
| CKAP2 | 1.2031 | [0.5052;1.9011] | 0.0007 |
| CKAP2L | 1.0197 | [0.1843;1.8551] | 0.0167 |
| CKAP4 | 1.4675 | [1.1079;1.8270] | <0.0001 |
| CKAP5 | 1.0474 | [0.4365;1.6583] | 0.0008 |
| CKMT1A | 0.6045 | [0.1658;1.0431] | 0.0069 |
| CKS1B | 1.2312 | [0.3962;2.0662] | 0.0039 |
| CKS2 | 0.8883 | [0.3010;1.4757] | 0.003 |
| CLASP1 | 1.4323 | [1.0898;1.7749] | <0.0001 |
| CLCN2 | 0.6461 | [0.0170;1.2753] | 0.0441 |
| CLDN1 | 1.2251 | [0.5462;1.9040] | 0.0004 |
| CLDN12 | 1.4229 | [0.5806;2.2651] | 0.0009 |
| CLDN14 | 0.3615 | [0.0540;0.6689] | 0.0212 |
| CLDN16 | 0.6845 | [0.1384;1.2306] | 0.014 |
| CLEC5A | 1.0225 | [0.6810;1.3639] | <0.0001 |
| CLEC6A | 0.6434 | [0.0667;1.2201] | 0.0288 |
| CLEC7A | 1.108 | [0.7556;1.4604] | <0.0001 |
| CLIC4 | 0.8776 | [0.5646;1.1906] | <0.0001 |
| CLIP2 | 0.9144 | [0.5772;1.2515] | <0.0001 |
| CLSTN2 | 0.5938 | [0.0639;1.1237] | 0.0281 |
| CNKSR3 | 0.938 | [0.0414;1.8346] | 0.0403 |
| CNN3 | 0.9455 | [0.2900;1.6009] | 0.0047 |
| CNTLN | 0.6836 | [0.3429;1.0243] | <0.0001 |
| CNTNAP2 | 1.333 | [0.7298;1.9362] | <0.0001 |
| COA1 | 0.8061 | [0.3479;1.2644] | 0.0006 |
| COL10A1 | 1.0641 | [0.7471;1.3812] | <0.0001 |
| COL12A1 | 0.6564 | [0.1001;1.2127] | 0.0207 |
| COL17A1 | 1.2652 | [0.6775;1.8529] | <0.0001 |
| COL18A1 | 0.7444 | [0.1086;1.3802] | 0.0217 |
| COL1A1 | 0.7509 | [0.1107;1.3910] | 0.0215 |
| COL1A2 | 0.6051 | [0.0753;1.1349] | 0.0252 |
| COL22A1 | 1.4924 | [0.8694;2.1154] | <0.0001 |
| COL27A1 | 0.842 | [0.0948;1.5891] | 0.0272 |
| COL2A1 | 0.3766 | [0.0472;0.7061] | 0.0251 |
| COL3A1 | 0.6889 | [0.1924;1.1853] | 0.0065 |
| COL4A1 | 1.347 | [1.0132;1.6808] | <0.0001 |
| COL4A2 | 1.039 | [0.3022;1.7757] | 0.0057 |
| COL4A5 | 1.4895 | [0.7235;2.2555] | 0.0001 |
| COL4A6 | 0.3933 | [0.0868;0.6998] | 0.0119 |
| COL5A1 | 1.3129 | [0.9873;1.6385] | <0.0001 |
| COL5A2 | 0.8994 | [0.3631;1.4357] | 0.001 |
| COL6A1 | 0.5203 | [0.2141;0.8264] | 0.0009 |
| COL6A3 | 0.524 | [0.2137;0.8343] | 0.0009 |
| COL6A6 | 0.5459 | [0.2073;0.8844] | 0.0016 |
| COL7A1 | 1.2446 | [0.9158;1.5734] | <0.0001 |
| COL8A1 | 0.7873 | [0.1777;1.3969] | 0.0114 |
| CORO1C | 1.0398 | [0.7159;1.3637] | <0.0001 |
| COX17 | 0.6045 | [0.2830;0.9259] | 0.0002 |
| COX5A | 0.9824 | [0.4289;1.5359] | 0.0005 |
| COX6A1 | 0.6423 | [0.0757;1.2088] | 0.0263 |
| COX7B | 0.7679 | [0.2016;1.3342] | 0.0079 |
| CPEB2 | 0.517 | [0.1700;0.8639] | 0.0035 |
| CPNE4 | 0.4762 | [0.1594;0.7930] | 0.0032 |
| CPOX | 1.3287 | [0.9990;1.6584] | <0.0001 |
| CPS1 | 1.0199 | [0.6976;1.3421] | <0.0001 |
| CPT1C | 0.9716 | [0.6089;1.3344] | <0.0001 |
| CRNKL1 | 0.7771 | [0.4417;1.1126] | <0.0001 |
| CROT | 0.8727 | [0.5700;1.1754] | <0.0001 |
| CSAG1 | 1.0804 | [0.1923;1.9685] | 0.0171 |
| CSAG3 | 1.196 | [0.5067;1.8852] | 0.0007 |
| CSE1L | 1.8263 | [1.4753;2.1773] | <0.0001 |
| CSF1 | 0.4983 | [0.1683;0.8283] | 0.0031 |
| CSRP1 | 0.6458 | [0.1362;1.1555] | 0.013 |
| CSRP2 | 0.4568 | [0.1496;0.7641] | 0.0036 |
| CST1 | 0.8189 | [0.4788;1.1591] | <0.0001 |
| CST2 | 0.6483 | [0.3136;0.9831] | 0.0001 |
| CSTA | 0.8089 | [0.1322;1.4857] | 0.0191 |
| CTBP2 | 0.7797 | [0.0318;1.5276] | 0.041 |
| CTDSPL | 0.3457 | [0.0368;0.6546] | 0.0283 |
| CTLA4 | 0.3546 | [0.0497;0.6594] | 0.0226 |
| CTNNA3 | 0.4957 | [0.1898;0.8016] | 0.0015 |
| CTNNBIP1 | 0.6466 | [0.0409;1.2524] | 0.0364 |
| CTNND1 | 1.4087 | [0.6555;2.1619] | 0.0002 |
| CTPS1 | 1.6137 | [0.4173;2.8100] | 0.0082 |
| CTSC | 0.6241 | [0.3100;0.9382] | <0.0001 |
| CTSL | 0.6872 | [0.2902;1.0842] | 0.0007 |
| CTTNBP2NL | 1.399 | [0.4557;2.3423] | 0.0037 |
| CXADR | 1.2535 | [0.4766;2.0304] | 0.0016 |
| CXADRP2 | 0.7971 | [0.2687;1.3256] | 0.0031 |
| CXCL10 | 1.8146 | [0.9434;2.6858] | <0.0001 |
| CXCL11 | 1.4454 | [0.7702;2.1205] | <0.0001 |
| CXCL2 | 0.6516 | [0.1404;1.1628] | 0.0125 |
| CXCL3 | 1.2195 | [0.8957;1.5433] | <0.0001 |
| CXCL5 | 0.4721 | [0.1435;0.8008] | 0.0049 |
| CXCL9 | 1.0815 | [0.7552;1.4079] | <0.0001 |
| CYC1 | 0.9379 | [0.6160;1.2598] | <0.0001 |
| CYCS | 1.2444 | [0.5387;1.9501] | 0.0005 |
| CYP27C1 | 0.9131 | [0.4953;1.3310] | <0.0001 |
| CYP3A5 | 0.7558 | [0.1723;1.3393] | 0.0111 |
| DAB2IP | 0.7796 | [0.0415;1.5178] | 0.0384 |
| DAPL1 | 1.3568 | [0.6981;2.0154] | <0.0001 |
| DBF4 | 1.1339 | [0.3067;1.9610] | 0.0072 |
| DBI | 0.8072 | [0.2324;1.3820] | 0.0059 |
| DCAF6 | 0.958 | [0.6148;1.3011] | <0.0001 |
| DCAKD | 0.5598 | [0.2237;0.8959] | 0.0011 |
| DCBLD2 | 1.1548 | [0.5247;1.7848] | 0.0003 |
| DDIAS | 0.8657 | [0.3333;1.3981] | 0.0014 |
| DDIT3 | 0.5936 | [0.2842;0.9029] | 0.0002 |
| DDR1 | 2.0514 | [0.6735;3.4292] | 0.0035 |
| DDX11 | 0.8084 | [0.1406;1.4763] | 0.0177 |
| DDX52 | 1.0144 | [0.6928;1.3360] | <0.0001 |
| DDX55 | 1.1359 | [0.3661;1.9057] | 0.0038 |
| DDX58 | 0.5766 | [0.2702;0.8829] | 0.0002 |
| DDX60 | 1.1238 | [0.5095;1.7382] | 0.0003 |
| DEFB4A | 1.2932 | [0.8044;1.7820] | <0.0001 |
| DENND1B | 0.4143 | [0.0901;0.7385] | 0.0123 |
| DEPDC1 | 0.9647 | [0.6443;1.2851] | <0.0001 |
| DEPDC1B | 1.0712 | [0.5047;1.6377] | 0.0002 |
| DEPDC7 | 0.4235 | [0.0789;0.7681] | 0.016 |
| DERA | 0.9264 | [0.5816;1.2712] | <0.0001 |
| DESI1 | 0.925 | [0.0118;1.8382] | 0.0471 |
| DFNB31 | 0.6568 | [0.0733;1.2404] | 0.0274 |
| DGAT2 | 1.3539 | [1.0114;1.6965] | <0.0001 |
| DGKZ | 0.4196 | [0.1153;0.7239] | 0.0069 |
| DHCR7 | 1.0375 | [0.4993;1.5756] | 0.0002 |
| DHFR | 0.9495 | [0.2673;1.6316] | 0.0064 |
| DHRS2 | 0.9936 | [0.6442;1.3429] | <0.0001 |
| DHRSX | 1.2831 | [0.9108;1.6553] | <0.0001 |
| DHX34 | 1.0838 | [0.3553;1.8124] | 0.0035 |
| DIRAS3 | 0.4666 | [0.1386;0.7947] | 0.0053 |
| DKC1 | 0.9968 | [0.2776;1.7159] | 0.0066 |
| DKK1 | 0.6587 | [0.3508;0.9667] | <0.0001 |
| DKK3 | 0.9737 | [0.6564;1.2910] | <0.0001 |
| DLD | 0.8977 | [0.5835;1.2119] | <0.0001 |
| DLG1 | 0.649 | [0.1037;1.1942] | 0.0197 |
| DLG3 | 1.3714 | [0.1092;2.6336] | 0.0332 |
| DLG5 | 1.4351 | [0.4129;2.4573] | 0.0059 |
| DLGAP5 | 1.3625 | [0.3047;2.4203] | 0.0116 |
| DLX4 | 0.9459 | [0.6230;1.2689] | <0.0001 |
| DLX5 | 0.5293 | [0.2258;0.8328] | 0.0006 |
| DLX6 | 0.5556 | [0.0587;1.0524] | 0.0284 |
| DMD | 0.9983 | [0.4000;1.5967] | 0.0011 |
| DMPK | 0.5252 | [0.1920;0.8584] | 0.002 |
| DMRTA2 | 1.1692 | [0.4157;1.9228] | 0.0024 |
| DMXL2 | 0.7948 | [0.4555;1.1342] | <0.0001 |
| DNA2 | 1.0133 | [0.6635;1.3631] | <0.0001 |
| DNAAF2 | 0.3576 | [0.0104;0.7047] | 0.0435 |
| DNAJA1 | 0.7326 | [0.3975;1.0677] | <0.0001 |
| DNAJB6 | 1.2069 | [0.4981;1.9156] | 0.0008 |
| DNAJC2 | 0.7204 | [0.0134;1.4274] | 0.0458 |
| DNAJC9 | 0.8284 | [0.0036;1.6533] | 0.049 |
| DNM1L | 1.2942 | [0.6697;1.9188] | <0.0001 |
| DNMT3B | 0.8512 | [0.5344;1.1680] | <0.0001 |
| DOC2B | 0.982 | [0.0746;1.8894] | 0.0339 |
| DOCK4 | 1.5157 | [0.9325;2.0990] | <0.0001 |
| DONSON | 0.9249 | [0.1588;1.6911] | 0.018 |
| DPP9 | 0.6557 | [0.0150;1.2965] | 0.0449 |
| DPY19L1 | 0.7745 | [0.4339;1.1150] | <0.0001 |
| DQX1 | 1.1303 | [0.4029;1.8578] | 0.0023 |
| DRAM1 | 1.3765 | [1.0120;1.7409] | <0.0001 |
| DSC2 | 1.0588 | [0.4909;1.6267] | 0.0003 |
| DSC3 | 0.7319 | [0.1671;1.2968] | 0.0111 |
| DSCAM | 0.6954 | [0.3810;1.0098] | <0.0001 |
| DSCC1 | 1.2337 | [0.5161;1.9514] | 0.0008 |
| DSE | 0.8728 | [0.5393;1.2062] | <0.0001 |
| DSG2 | 1.9507 | [1.2781;2.6233] | <0.0001 |
| DSG3 | 0.8417 | [0.2409;1.4425] | 0.006 |
| DSN1 | 0.9531 | [0.2884;1.6179] | 0.005 |
| DSP | 1.5065 | [0.4739;2.5390] | 0.0042 |
| DST | 0.9572 | [0.6492;1.2651] | <0.0001 |
| DTL | 1.956 | [1.1329;2.7790] | <0.0001 |
| DTX3L | 1.4068 | [0.4349;2.3787] | 0.0046 |
| DTYMK | 0.9994 | [0.4519;1.5470] | 0.0003 |
| DUSP10 | 1.0499 | [0.4319;1.6679] | 0.0009 |
| DUSP16 | 1.0918 | [0.2558;1.9278] | 0.0105 |
| DUT | 0.9683 | [0.6494;1.2872] | <0.0001 |
| DYRK3 | 0.9571 | [0.2634;1.6508] | 0.0068 |
| DYSF | 0.3466 | [0.0420;0.6512] | 0.0257 |
| E2F6 | 1.3456 | [0.5815;2.1098] | 0.0006 |
| E2F7 | 0.8697 | [0.2374;1.5021] | 0.007 |
| EARS2 | 1.0989 | [0.2828;1.9150] | 0.0083 |
| ECT2 | 1.7459 | [1.1379;2.3540] | <0.0001 |
| EDARADD | 1.9445 | [0.8162;3.0728] | 0.0007 |
| EDIL3 | 0.3277 | [0.0210;0.6345] | 0.0363 |
| EDN2 | 0.7617 | [0.0312;1.4922] | 0.041 |
| EEA1 | 0.4599 | [0.1509;0.7688] | 0.0035 |
| EEPD1 | 0.5036 | [0.1543;0.8529] | 0.0047 |
| EFNA1 | 1.1008 | [0.3094;1.8921] | 0.0064 |
| EFNB1 | 0.9949 | [0.1236;1.8663] | 0.0252 |
| EFNB2 | 0.6216 | [0.3122;0.9311] | <0.0001 |
| EFS | 1.0125 | [0.4649;1.5601] | 0.0003 |
| EGFL6 | 1.1032 | [0.7798;1.4265] | <0.0001 |
| EGFR | 1.1918 | [0.1819;2.2017] | 0.0207 |
| EHBP1 | 1.061 | [0.4838;1.6381] | 0.0003 |
| EHHADH | 0.6062 | [0.0794;1.1331] | 0.0241 |
| EIF1AY | 0.4372 | [0.1241;0.7504] | 0.0062 |
| EIF2AK2 | 1.3505 | [0.5724;2.1285] | 0.0007 |
| EIF2S2 | 0.9437 | [0.3386;1.5488] | 0.0022 |
| EIF3C | 0.3674 | [0.0289;0.7059] | 0.0334 |
| EIF4E | 0.8255 | [0.4922;1.1588] | <0.0001 |
| EIF4EBP1 | 0.9787 | [0.2537;1.7037] | 0.0082 |
| ELAVL2 | 0.3404 | [0.0402;0.6405] | 0.0262 |
| ELOVL7 | 1.154 | [0.7942;1.5138] | <0.0001 |
| EME1 | 1.2529 | [0.3768;2.1290] | 0.0051 |
| EMILIN2 | 0.7455 | [0.2352;1.2557] | 0.0042 |
| EMP2 | 0.7849 | [0.0746;1.4953] | 0.0303 |
| ENAH | 1.2057 | [0.5391;1.8722] | 0.0004 |
| ENC1 | 0.3744 | [0.0689;0.6799] | 0.0163 |
| ENO1 | 0.8596 | [0.1718;1.5474] | 0.0143 |
| ENOX1 | 0.533 | [0.2029;0.8631] | 0.0016 |
| ENPP6 | 0.4331 | [0.1228;0.7434] | 0.0062 |
| EPCAM | 1.6735 | [0.8264;2.5207] | 0.0001 |
| EPHB2 | 1.123 | [0.4931;1.7529] | 0.0005 |
| EPHB4 | 1.6125 | [0.8299;2.3952] | <0.0001 |
| EPSTI1 | 0.7312 | [0.1818;1.2807] | 0.0091 |
| ERAP1 | 0.6401 | [0.3090;0.9711] | 0.0002 |
| ERC2 | 0.8517 | [0.5098;1.1935] | <0.0001 |
| ERCC6L | 0.9027 | [0.3161;1.4892] | 0.0026 |
| ERI1 | 1.0004 | [0.6408;1.3599] | <0.0001 |
| ERI2 | 1.2648 | [0.6100;1.9196] | 0.0002 |
| ERO1LB | 1.0081 | [0.4240;1.5921] | 0.0007 |
| ERRFI1 | 0.944 | [0.1716;1.7164] | 0.0166 |
| ESCO2 | 1.6271 | [0.7059;2.5483] | 0.0005 |
| ESM1 | 1.1711 | [0.6480;1.6942] | <0.0001 |
| ESPN | 0.6354 | [0.0447;1.2261] | 0.035 |
| ESRP1 | 1.277 | [0.0564;2.4976] | 0.0403 |
| ESRRG | 0.4325 | [0.1264;0.7386] | 0.0056 |
| ETAA1 | 0.3692 | [0.0388;0.6996] | 0.0285 |
| ETV6 | 0.9284 | [0.3602;1.4965] | 0.0014 |
| ETV7 | 1.3303 | [0.6495;2.0112] | 0.0001 |
| EVPLL | 0.4617 | [0.1084;0.8150] | 0.0104 |
| EXO1 | 1.449 | [0.7999;2.0981] | <0.0001 |
| EXT1 | 1.1151 | [0.5820;1.6481] | <0.0001 |
| EXTL3 | 0.6557 | [0.0934;1.2180] | 0.0223 |
| EZH2 | 1.0928 | [0.0395;2.1461] | 0.042 |
| F11R | 1.1965 | [0.2452;2.1478] | 0.0137 |
| F2R | 0.7524 | [0.4337;1.0711] | <0.0001 |
| F2RL3 | 0.4205 | [0.1160;0.7250] | 0.0068 |
| FABP6 | 0.8961 | [0.4054;1.3867] | 0.0003 |
| FADD | 0.4113 | [0.1030;0.7196] | 0.0089 |
| FADS1 | 0.9324 | [0.4634;1.4013] | <0.0001 |
| FAF1 | 0.7016 | [0.3883;1.0150] | <0.0001 |
| FAM111B | 0.9479 | [0.0660;1.8297] | 0.0351 |
| FAM135A | 0.8068 | [0.1980;1.4156] | 0.0094 |
| FAM156B | 1.1599 | [0.5237;1.7960] | 0.0004 |
| FAM160A1 | 1.0849 | [0.0393;2.1304] | 0.042 |
| FAM161A | 0.4802 | [0.1399;0.8204] | 0.0057 |
| FAM171A1 | 1.1553 | [0.3279;1.9827] | 0.0062 |
| FAM189B | 1.2809 | [0.6144;1.9474] | 0.0002 |
| FAM20B | 0.7205 | [0.4092;1.0318] | <0.0001 |
| FAM222A | 1.2017 | [0.6927;1.7107] | <0.0001 |
| FAM57A | 1.2054 | [0.3029;2.1078] | 0.0089 |
| FAM64A | 1.6528 | [1.2502;2.0555] | <0.0001 |
| FAM71E1 | 0.7781 | [0.0358;1.5204] | 0.0399 |
| FAM71F1 | 0.8099 | [0.1529;1.4669] | 0.0157 |
| FAM83B | 1.7809 | [0.8585;2.7033] | 0.0002 |
| FAM83D | 1.0624 | [0.1973;1.9276] | 0.0161 |
| FAM84A | 1.0882 | [0.2616;1.9148] | 0.0099 |
| FAM84B | 1.2064 | [0.2071;2.2057] | 0.018 |
| FAM86B1 | 0.9608 | [0.3680;1.5535] | 0.0015 |
| FAM86B2 | 0.6296 | [0.1866;1.0727] | 0.0053 |
| FANCB | 1.0043 | [0.3875;1.6211] | 0.0014 |
| FANCC | 0.6717 | [0.3608;0.9826] | <0.0001 |
| FANCI | 1.5122 | [0.7375;2.2868] | 0.0001 |
| FANCL | 1.6923 | [0.9899;2.3948] | <0.0001 |
| FAP | 0.8131 | [0.1322;1.4940] | 0.0193 |
| FAS | 0.8314 | [0.4768;1.1859] | <0.0001 |
| FASLG | 0.6939 | [0.3611;1.0267] | <0.0001 |
| FAT1 | 1.1327 | [0.3040;1.9613] | 0.0074 |
| FAT2 | 1.0181 | [0.2310;1.8051] | 0.0112 |
| FBN2 | 0.3562 | [0.0518;0.6607] | 0.0218 |
| FBXL4 | 0.6253 | [0.1217;1.1289] | 0.015 |
| FBXO21 | 0.574 | [0.2668;0.8812] | 0.0003 |
| FBXO27 | 1.0369 | [0.3689;1.7049] | 0.0023 |
| FBXO39 | 0.8304 | [0.4814;1.1793] | <0.0001 |
| FBXO5 | 0.8464 | [0.1812;1.5117] | 0.0126 |
| FCGBP | 0.507 | [0.2054;0.8086] | 0.001 |
| FCGR1A | 0.712 | [0.3476;1.0765] | 0.0001 |
| FCGR1B | 0.8406 | [0.1635;1.5178] | 0.015 |
| FCGR3A | 1.2359 | [0.8213;1.6505] | <0.0001 |
| FCGR3B | 1.0568 | [0.6897;1.4240] | <0.0001 |
| FEN1 | 0.8129 | [0.1398;1.4860] | 0.0179 |
| FERMT1 | 2.268 | [1.2696;3.2663] | <0.0001 |
| FGD1 | 0.8474 | [0.1353;1.5594] | 0.0197 |
| FGD6 | 1.3774 | [1.0131;1.7416] | <0.0001 |
| FGF1 | 1.1584 | [0.5332;1.7836] | 0.0003 |
| FGF17 | 0.5315 | [0.0270;1.0361] | 0.039 |
| FGF2 | 0.8213 | [0.5110;1.1316] | <0.0001 |
| FGFR3 | 0.9969 | [0.1322;1.8617] | 0.0239 |
| FHOD3 | 1.158 | [0.8109;1.5052] | <0.0001 |
| FIGNL1 | 1.0971 | [0.3954;1.7988] | 0.0022 |
| FJX1 | 1.9815 | [1.5875;2.3754] | <0.0001 |
| FKBP4 | 1.4718 | [0.9212;2.0223] | <0.0001 |
| FKTN | 0.4858 | [0.1483;0.8234] | 0.0048 |
| FLNA | 0.4128 | [0.1065;0.7190] | 0.0083 |
| FLVCR1 | 1.0001 | [0.6371;1.3632] | <0.0001 |
| FMNL2 | 1.4736 | [1.1226;1.8247] | <0.0001 |
| FN1 | 1.4874 | [1.1459;1.8289] | <0.0001 |
| FNBP1L | 1.2378 | [0.5434;1.9323] | 0.0005 |
| FNDC3B | 1.7616 | [0.5211;3.0021] | 0.0054 |
| FOSL2 | 1.2428 | [0.4529;2.0328] | 0.002 |
| FOXI1 | 0.5176 | [0.2100;0.8252] | 0.001 |
| FOXK1 | 0.8911 | [0.1842;1.5980] | 0.0135 |
| FOXM1 | 1.7645 | [1.0628;2.4661] | <0.0001 |
| FOXN1 | 0.9981 | [0.4312;1.5650] | 0.0006 |
| FOXQ1 | 0.8972 | [0.1184;1.6759] | 0.0239 |
| FOXRED2 | 1.0632 | [0.4606;1.6659] | 0.0005 |
| FPR3 | 1.3254 | [0.7472;1.9035] | <0.0001 |
| FRAS1 | 1.0139 | [0.6715;1.3563] | <0.0001 |
| FRK | 1.0055 | [0.2518;1.7593] | 0.0089 |
| FRMD4A | 1.1339 | [0.5123;1.7555] | 0.0003 |
| FRRS1 | 1.413 | [0.4820;2.3441] | 0.0029 |
| FSCN1 | 1.1028 | [0.3814;1.8243] | 0.0027 |
| FST | 0.4922 | [0.1832;0.8011] | 0.0018 |
| FSTL3 | 0.5774 | [0.2658;0.8891] | 0.0003 |
| FSTL4 | 0.7977 | [0.1718;1.4236] | 0.0125 |
| FTH1 | 0.9564 | [0.6375;1.2752] | <0.0001 |
| FUT1 | 0.9892 | [0.2267;1.7517] | 0.011 |
| FUT10 | 0.9986 | [0.6649;1.3323] | <0.0001 |
| FXYD2 | 0.6184 | [0.3081;0.9286] | <0.0001 |
| FZD6 | 1.3701 | [0.7425;1.9977] | <0.0001 |
| FZD7 | 1.4402 | [1.1092;1.7712] | <0.0001 |
| FZD8 | 0.8419 | [0.0996;1.5843] | 0.0262 |
| GAA | 0.8123 | [0.4738;1.1508] | <0.0001 |
| GABPB1 | 1.0385 | [0.6930;1.3840] | <0.0001 |
| GABRE | 0.5906 | [0.1973;0.9839] | 0.0033 |
| GABRG3 | 0.4597 | [0.1525;0.7670] | 0.0034 |
| GABRQ | 0.4668 | [0.1568;0.7768] | 0.0032 |
| GAD1 | 1.4121 | [0.8672;1.9570] | <0.0001 |
| GADD45A | 1.5492 | [1.0234;2.0750] | <0.0001 |
| GALNT11 | 1.8268 | [1.4813;2.1723] | <0.0001 |
| GALNT2 | 1.0414 | [0.6892;1.3937] | <0.0001 |
| GAPDH | 1.3802 | [0.5991;2.1614] | 0.0005 |
| GART | 1.2489 | [0.9180;1.5798] | <0.0001 |
| GAS2L3 | 1.2484 | [0.8802;1.6167] | <0.0001 |
| GAS6 | 0.4595 | [0.1528;0.7662] | 0.0033 |
| GATA6 | 0.9273 | [0.3608;1.4938] | 0.0013 |
| GBP1 | 0.8512 | [0.2904;1.4120] | 0.0029 |
| GBP4 | 0.6319 | [0.0175;1.2463] | 0.0438 |
| GCSH | 0.7531 | [0.1402;1.3660] | 0.016 |
| GEMIN8P4 | 1.0956 | [0.5636;1.6276] | <0.0001 |
| GFM1 | 1.0553 | [0.7100;1.4007] | <0.0001 |
| GFPT2 | 0.7859 | [0.4713;1.1006] | <0.0001 |
| GGCX | 0.736 | [0.1378;1.3342] | 0.0159 |
| GGH | 1.0763 | [0.7530;1.3995] | <0.0001 |
| GGT8P | 1.0503 | [0.5015;1.5991] | 0.0002 |
| GHR | 0.8474 | [0.5355;1.1593] | <0.0001 |
| GINS1 | 2.2383 | [1.0759;3.4007] | 0.0002 |
| GINS2 | 0.9465 | [0.0525;1.8405] | 0.038 |
| GINS3 | 1.2942 | [0.4334;2.1550] | 0.0032 |
| GINS4 | 1.0175 | [0.2503;1.7847] | 0.0093 |
| GJA1 | 1.4933 | [1.1629;1.8238] | <0.0001 |
| GJB3 | 1.525 | [0.2723;2.7776] | 0.017 |
| GJB5 | 1.6758 | [0.5129;2.8386] | 0.0047 |
| GLCE | 1.5128 | [0.7552;2.2704] | <0.0001 |
| GLRX3 | 0.7534 | [0.4157;1.0912] | <0.0001 |
| GLS2 | 1.1963 | [0.6167;1.7759] | <0.0001 |
| GLTP | 1.3148 | [0.4098;2.2198] | 0.0044 |
| GMFB | 0.6771 | [0.3669;0.9873] | <0.0001 |
| GMNN | 1.2162 | [0.8890;1.5434] | <0.0001 |
| GNA15 | 1.0132 | [0.1300;1.8963] | 0.0245 |
| GNG12 | 0.9747 | [0.1513;1.7981] | 0.0203 |
| GNL2 | 1.2721 | [0.9112;1.6331] | <0.0001 |
| GNLY | 0.688 | [0.1188;1.2573] | 0.0178 |
| GNPDA1 | 1.2252 | [0.6631;1.7874] | <0.0001 |
| GOLIM4 | 0.6386 | [0.3062;0.9711] | 0.0002 |
| GORAB | 1.3697 | [0.6438;2.0957] | 0.0002 |
| GPC1 | 1.0347 | [0.2494;1.8199] | 0.0098 |
| GPC4 | 0.6096 | [0.0507;1.1684] | 0.0325 |
| GPHN | 0.642 | [0.0845;1.1995] | 0.024 |
| GPI | 1.2096 | [0.5093;1.9098] | 0.0007 |
| GPNMB | 0.9057 | [0.5860;1.2254] | <0.0001 |
| GPR107 | 1.3941 | [0.7272;2.0611] | <0.0001 |
| GPR125 | 1.4925 | [0.4467;2.5384] | 0.0052 |
| GPR128 | 0.689 | [0.3401;1.0379] | 0.0001 |
| GPR141 | 0.8977 | [0.4317;1.3636] | 0.0002 |
| GPR143 | 0.9506 | [0.4320;1.4691] | 0.0003 |
| GPR161 | 1.0148 | [0.3434;1.6862] | 0.0031 |
| GPR37 | 0.6658 | [0.1328;1.1989] | 0.0144 |
| GPR39 | 0.7826 | [0.2213;1.3438] | 0.0063 |
| GPR50 | 0.3335 | [0.0301;0.6370] | 0.0312 |
| GPR84 | 0.5846 | [0.2660;0.9033] | 0.0003 |
| GPR87 | 1.1089 | [0.2504;1.9674] | 0.0114 |
| GPRIN1 | 0.8805 | [0.1731;1.5880] | 0.0147 |
| GPSM2 | 1.4984 | [0.7938;2.2030] | <0.0001 |
| GRB10 | 1.4342 | [1.1001;1.7683] | <0.0001 |
| GRB14 | 0.5032 | [0.1983;0.8082] | 0.0012 |
| GRB7 | 0.7246 | [0.0694;1.3798] | 0.0302 |
| GREM1 | 0.8553 | [0.5374;1.1733] | <0.0001 |
| GRHL2 | 1.3818 | [0.2732;2.4904] | 0.0146 |
| GRIN2A | 0.5271 | [0.1957;0.8585] | 0.0018 |
| GRIP1 | 0.5158 | [0.2134;0.8182] | 0.0008 |
| GRTP1 | 1.082 | [0.1516;2.0123] | 0.0226 |
| GSTCD | 0.928 | [0.2723;1.5837] | 0.0055 |
| GSTP1 | 0.7595 | [0.0150;1.5039] | 0.0455 |
| GTF2F2 | 0.9817 | [0.6385;1.3250] | <0.0001 |
| GTF2H3 | 1.3132 | [0.7203;1.9061] | <0.0001 |
| GTF2I | 0.8121 | [0.4964;1.1278] | <0.0001 |
| GTF2IRD1 | 1.091 | [0.1622;2.0199] | 0.0213 |
| GUCY1A2 | 0.355 | [0.0530;0.6570] | 0.0212 |
| GZMB | 0.5262 | [0.2214;0.8309] | 0.0007 |
| GZMH | 0.6312 | [0.3215;0.9409] | <0.0001 |
| H1FX | 0.9638 | [0.2209;1.7067] | 0.011 |
| H2AFY | 0.7241 | [0.0382;1.4100] | 0.0385 |
| H2AFY2 | 0.6904 | [0.0440;1.3369] | 0.0363 |
| H2AFZ | 1.2428 | [0.9040;1.5817] | <0.0001 |
| HAPLN1 | 0.7444 | [0.4281;1.0607] | <0.0001 |
| HAPLN3 | 0.6578 | [0.3116;1.0041] | 0.0002 |
| HAUS1 | 0.8325 | [0.1274;1.5376] | 0.0207 |
| HAUS6 | 0.8172 | [0.4777;1.1568] | <0.0001 |
| HAVCR2 | 0.4997 | [0.1827;0.8167] | 0.002 |
| HCAR2 | 0.7451 | [0.2035;1.2867] | 0.007 |
| HCAR3 | 0.7531 | [0.3954;1.1108] | <0.0001 |
| HCN3 | 0.6367 | [0.0441;1.2294] | 0.0352 |
| HCRTR2 | 0.3477 | [0.0449;0.6504] | 0.0244 |
| HDAC2 | 1.4388 | [1.1074;1.7702] | <0.0001 |
| HDGF | 1.1215 | [0.2381;2.0050] | 0.0128 |
| HDGFRP3 | 1.9342 | [1.5145;2.3540] | <0.0001 |
| HDX | 0.7922 | [0.4432;1.1413] | <0.0001 |
| HELLS | 1.0252 | [0.2486;1.8018] | 0.0097 |
| HERC5 | 0.7243 | [0.4151;1.0334] | <0.0001 |
| HES6 | 0.5729 | [0.2545;0.8912] | 0.0004 |
| HILPDA | 0.9636 | [0.6066;1.3205] | <0.0001 |
| HIP1 | 0.7471 | [0.4325;1.0618] | <0.0001 |
| HIST1H2AI | 2.4912 | [1.2352;3.7473] | 0.0001 |
| HIST1H4G | 0.4028 | [0.0352;0.7703] | 0.0317 |
| HIST2H2AA4 | 1.3434 | [0.8320;1.8548] | <0.0001 |
| HIST2H3A | 1.8585 | [0.3788;3.3382] | 0.0138 |
| HIST2H4B | 1.6997 | [0.5203;2.8791] | 0.0047 |
| HJURP | 1.4606 | [0.4233;2.4979] | 0.0058 |
| HLTF | 0.7596 | [0.4211;1.0982] | <0.0001 |
| HMGA2 | 0.864 | [0.5186;1.2095] | <0.0001 |
| HMGCR | 0.8224 | [0.5139;1.1308] | <0.0001 |
| HMGCS1 | 0.5301 | [0.1940;0.8663] | 0.002 |
| HNF4G | 0.4278 | [0.1226;0.7330] | 0.006 |
| HNRNPAB | 1.0644 | [0.4116;1.7172] | 0.0014 |
| HOMER1 | 1.074 | [0.5374;1.6106] | <0.0001 |
| HOMER3 | 1.16 | [0.5626;1.7574] | 0.0001 |
| HOOK1 | 0.747 | [0.0390;1.4550] | 0.0387 |
| HOOK2 | 1.2064 | [0.5291;1.8836] | 0.0005 |
| HOXA10 | 1.7632 | [1.1731;2.3534] | <0.0001 |
| HOXA3 | 0.8302 | [0.1260;1.5343] | 0.0208 |
| HOXA4 | 0.838 | [0.5217;1.1542] | <0.0001 |
| HOXA7 | 1.0177 | [0.6847;1.3507] | <0.0001 |
| HOXA9 | 1.0393 | [0.1626;1.9160] | 0.0202 |
| HOXB13 | 1.1732 | [0.3207;2.0258] | 0.007 |
| HOXB9 | 0.8629 | [0.3335;1.3923] | 0.0014 |
| HOXC13 | 0.8055 | [0.0403;1.5708] | 0.0391 |
| HOXC6 | 0.9543 | [0.2010;1.7075] | 0.013 |
| HOXC8 | 1.0003 | [0.2807;1.7198] | 0.0064 |
| HOXC9 | 1.0805 | [0.0806;2.0803] | 0.0342 |
| HPRT1 | 0.9464 | [0.6246;1.2681] | <0.0001 |
| HR | 0.6668 | [0.1599;1.1737] | 0.0099 |
| HRAS | 0.7241 | [0.2040;1.2443] | 0.0064 |
| HS3ST4 | 1.2723 | [0.9102;1.6343] | <0.0001 |
| HS6ST1 | 1.4942 | [0.6355;2.3529] | 0.0006 |
| HSD17B6 | 0.6785 | [0.1066;1.2505] | 0.0201 |
| HSPA1A | 1.1278 | [0.1096;2.1460] | 0.0299 |
| HSPA4L | 1.7173 | [0.8514;2.5832] | 0.0001 |
| HSPB1 | 0.784 | [0.0919;1.4761] | 0.0264 |
| HSPD1 | 1.4014 | [0.8198;1.9829] | <0.0001 |
| HSPE1 | 1.4358 | [1.0937;1.7779] | <0.0001 |
| HTATIP2 | 0.4378 | [0.1295;0.7460] | 0.0054 |
| HTRA3 | 0.5534 | [0.2087;0.8981] | 0.0017 |
| IBSP | 0.6915 | [0.3806;1.0023] | <0.0001 |
| ICAM1 | 1.1487 | [0.7971;1.5004] | <0.0001 |
| ICAM5 | 0.7951 | [0.2094;1.3809] | 0.0078 |
| ICOSLG | 1.0605 | [0.7236;1.3974] | <0.0001 |
| ID2 | 0.9788 | [0.3630;1.5946] | 0.0018 |
| IDH1 | 1.9208 | [1.3225;2.5190] | <0.0001 |
| IDO1 | 0.7584 | [0.1551;1.3617] | 0.0138 |
| IER5L | 1.084 | [0.3136;1.8544] | 0.0058 |
| IFI27 | 1.0205 | [0.0986;1.9425] | 0.03 |
| IFI44 | 1.1607 | [0.6101;1.7113] | <0.0001 |
| IFI44L | 1.0507 | [0.7070;1.3943] | <0.0001 |
| IFI6 | 1.0451 | [0.3464;1.7437] | 0.0034 |
| IFIH1 | 1.1881 | [0.5028;1.8734] | 0.0007 |
| IFIT1 | 1.0551 | [0.7128;1.3973] | <0.0001 |
| IFIT2 | 0.7556 | [0.4198;1.0913] | <0.0001 |
| IFIT3 | 1.2401 | [0.9138;1.5663] | <0.0001 |
| IFNG | 0.6483 | [0.0430;1.2536] | 0.0358 |
| IFRD1 | 1.0435 | [0.7205;1.3665] | <0.0001 |
| IGF1R | 0.6887 | [0.0697;1.3076] | 0.0292 |
| IGF2BP3 | 1.3641 | [0.4241;2.3041] | 0.0045 |
| IGF2R | 0.9543 | [0.6339;1.2748] | <0.0001 |
| IGFBP2 | 0.9145 | [0.1472;1.6818] | 0.0195 |
| IGFL2 | 0.3513 | [0.0128;0.6899] | 0.0419 |
| IGSF1 | 0.5925 | [0.0825;1.1024] | 0.0228 |
| IGSF3 | 1.4996 | [0.7209;2.2782] | 0.0002 |
| IGSF6 | 0.3107 | [0.0075;0.6138] | 0.0446 |
| IGSF9 | 1.1692 | [0.0337;2.3048] | 0.0436 |
| IKBKE | 0.5806 | [0.0231;1.1381] | 0.0412 |
| IL12RB2 | 0.5602 | [0.2539;0.8666] | 0.0003 |
| IL13RA1 | 0.6798 | [0.0146;1.3449] | 0.0452 |
| IL13RA2 | 0.9276 | [0.6118;1.2434] | <0.0001 |
| IL15 | 0.7756 | [0.1415;1.4097] | 0.0165 |
| IL15RA | 0.9246 | [0.6150;1.2341] | <0.0001 |
| IL17RB | 0.3797 | [0.0736;0.6859] | 0.0151 |
| IL1A | 0.9869 | [0.6661;1.3078] | <0.0001 |
| IL1B | 0.6198 | [0.3134;0.9263] | <0.0001 |
| IL1RAP | 0.6708 | [0.1367;1.2050] | 0.0138 |
| IL23A | 1.109 | [0.7892;1.4287] | <0.0001 |
| IL31RA | 0.6503 | [0.3285;0.9720] | <0.0001 |
| IL32 | 0.5329 | [0.1970;0.8688] | 0.0019 |
| IL36G | 0.481 | [0.1276;0.8344] | 0.0076 |
| IMPACT | 1.4309 | [1.0780;1.7838] | <0.0001 |
| INHBA | 1.0615 | [0.4316;1.6915] | 0.001 |
| INPP1 | 1.3031 | [0.7852;1.8209] | <0.0001 |
| INPP5J | 0.8881 | [0.2812;1.4950] | 0.0041 |
| INSM1 | 1.1298 | [0.8056;1.4539] | <0.0001 |
| INTS12 | 0.6382 | [0.3053;0.9712] | 0.0002 |
| INTS7 | 1.1029 | [0.3181;1.8877] | 0.0059 |
| IPO5 | 0.8182 | [0.1389;1.4974] | 0.0182 |
| IQCB1 | 0.3741 | [0.0417;0.7066] | 0.0274 |
| IQCJ-SCHIP1 | 1.3294 | [0.6373;2.0215] | 0.0002 |
| IREB2 | 0.5907 | [0.2896;0.8919] | 0.0001 |
| IRF1 | 0.6547 | [0.0147;1.2947] | 0.045 |
| IRF6 | 1.227 | [0.0289;2.4252] | 0.0447 |
| IRX2 | 0.7722 | [0.4292;1.1151] | <0.0001 |
| IRX6 | 1.0329 | [0.5829;1.4829] | <0.0001 |
| ISG15 | 0.8728 | [0.5350;1.2105] | <0.0001 |
| ISYNA1 | 1.2003 | [0.3182;2.0825] | 0.0077 |
| ITGA1 | 0.5949 | [0.0002;1.1897] | 0.0499 |
| ITGA3 | 0.7931 | [0.0561;1.5301] | 0.0349 |
| ITGA6 | 0.7869 | [0.0582;1.5156] | 0.0343 |
| ITGAE | 0.8577 | [0.5445;1.1708] | <0.0001 |
| ITGAV | 2.0195 | [1.6624;2.3767] | <0.0001 |
| ITGB1 | 0.9044 | [0.5643;1.2446] | <0.0001 |
| ITGB3BP | 0.5566 | [0.2478;0.8654] | 0.0004 |
| ITGB4 | 0.8011 | [0.0809;1.5213] | 0.0292 |
| ITGB6 | 1.7564 | [0.6635;2.8493] | 0.0016 |
| ITGB8 | 1.2523 | [0.3125;2.1920] | 0.009 |
| ITPKA | 0.8148 | [0.2207;1.4090] | 0.0072 |
| ITSN1 | 0.9085 | [0.2205;1.5964] | 0.0096 |
| IZUMO1 | 0.5998 | [0.0274;1.1721] | 0.04 |
| JAG1 | 0.9494 | [0.1804;1.7184] | 0.0155 |
| JAG2 | 1.1667 | [0.5295;1.8039] | 0.0003 |
| JAK2 | 0.5928 | [0.2594;0.9263] | 0.0005 |
| JAKMIP1 | 0.7402 | [0.3904;1.0900] | <0.0001 |
| JMJD4 | 0.7806 | [0.1122;1.4490] | 0.0221 |
| JPH1 | 0.8645 | [0.2072;1.5219] | 0.0099 |
| JUN | 0.7675 | [0.1221;1.4129] | 0.0198 |
| JUP | 1.1173 | [0.1050;2.1297] | 0.0305 |
| KCNB2 | 0.9052 | [0.0751;1.7354] | 0.0326 |
| KCNH2 | 0.4453 | [0.1401;0.7505] | 0.0042 |
| KCNJ6 | 0.4499 | [0.1441;0.7557] | 0.0039 |
| KCNK1 | 0.8642 | [0.3205;1.4080] | 0.0018 |
| KCNMA1 | 0.8806 | [0.5727;1.1884] | <0.0001 |
| KCNMB4 | 0.4505 | [0.1491;0.7520] | 0.0034 |
| KCNS3 | 0.8053 | [0.2876;1.3230] | 0.0023 |
| KCTD13 | 0.4819 | [0.1471;0.8167] | 0.0048 |
| KCTD3 | 1.8763 | [1.5265;2.2262] | <0.0001 |
| KDELC2 | 0.3956 | [0.0428;0.7485] | 0.028 |
| KDELR2 | 0.9204 | [0.1782;1.6626] | 0.0151 |
| KDM2B | 0.5662 | [0.2191;0.9134] | 0.0014 |
| KDM4D | 0.3291 | [0.0031;0.6550] | 0.0479 |
| KDM5B | 1.2872 | [0.6052;1.9692] | 0.0002 |
| KDSR | 0.9437 | [0.6013;1.2861] | <0.0001 |
| KEAP1 | 1.5067 | [0.6996;2.3137] | 0.0003 |
| KHK | 0.4072 | [0.0992;0.7153] | 0.0096 |
| KHSRP | 1.2192 | [0.5786;1.8597] | 0.0002 |
| KIAA0101 | 1.0851 | [0.7327;1.4375] | <0.0001 |
| KIAA1147 | 0.4493 | [0.0931;0.8056] | 0.0134 |
| KIAA1217 | 0.8653 | [0.2803;1.4502] | 0.0037 |
| KIAA1467 | 0.4617 | [0.0901;0.8334] | 0.0149 |
| KIAA1598 | 0.8586 | [0.5107;1.2066] | <0.0001 |
| KIF11 | 1.5602 | [0.6260;2.4943] | 0.0011 |
| KIF13A | 1.3407 | [0.4973;2.1842] | 0.0018 |
| KIF14 | 1.4605 | [0.8229;2.0981] | <0.0001 |
| KIF15 | 1.1922 | [0.3545;2.0298] | 0.0053 |
| KIF18A | 1.4483 | [0.5893;2.3073] | 0.001 |
| KIF18B | 1.1704 | [0.2624;2.0784] | 0.0115 |
| KIF20A | 1.2846 | [0.5451;2.0241] | 0.0007 |
| KIF20B | 0.8333 | [0.0341;1.6325] | 0.041 |
| KIF23 | 1.8351 | [1.0172;2.6531] | <0.0001 |
| KIF2C | 1.0616 | [0.3786;1.7446] | 0.0023 |
| KIF4A | 1.277 | [0.5725;1.9814] | 0.0004 |
| KITLG | 1.2132 | [0.5759;1.8506] | 0.0002 |
| KLF11 | 0.9817 | [0.6420;1.3215] | <0.0001 |
| KLHL1 | 0.4919 | [0.1632;0.8206] | 0.0034 |
| KLHL23 | 0.8929 | [0.0281;1.7577] | 0.043 |
| KLHL5 | 0.8019 | [0.1083;1.4954] | 0.0234 |
| KLRAP1 | 0.5607 | [0.1873;0.9341] | 0.0032 |
| KLRC2 | 0.7848 | [0.3657;1.2040] | 0.0002 |
| KLRG2 | 2.4554 | [1.6668;3.2440] | <0.0001 |
| KNTC1 | 1.3855 | [0.6775;2.0934] | 0.0001 |
| KPNA2 | 1.2714 | [0.5238;2.0190] | 0.0009 |
| KREMEN1 | 1.3023 | [0.2885;2.3162] | 0.0118 |
| KREMEN2 | 1.781 | [1.4348;2.1271] | <0.0001 |
| KRT16 | 0.8136 | [0.4735;1.1537] | <0.0001 |
| KRT16P3 | 0.8804 | [0.3573;1.4035] | 0.001 |
| KRT17 | 0.9651 | [0.5739;1.3563] | <0.0001 |
| KRT32 | 0.6316 | [0.2977;0.9655] | 0.0002 |
| KRT5 | 1.0746 | [0.2935;1.8557] | 0.007 |
| KRTCAP3 | 1.2044 | [0.3431;2.0658] | 0.0061 |
| KSR1 | 0.6127 | [0.0155;1.2100] | 0.0444 |
| KTN1-AS1 | 1.5797 | [0.4691;2.6903] | 0.0053 |
| KYNU | 0.4519 | [0.1467;0.7571] | 0.0037 |
| L3MBTL4 | 1.786 | [0.7891;2.7830] | 0.0004 |
| LACTB2 | 0.6029 | [0.2942;0.9115] | 0.0001 |
| LAIR2 | 0.3999 | [0.0944;0.7054] | 0.0103 |
| LAMA1 | 1.0798 | [0.7316;1.4280] | <0.0001 |
| LAMA3 | 0.8303 | [0.5215;1.1391] | <0.0001 |
| LAMB1 | 1.5464 | [0.9211;2.1718] | <0.0001 |
| LAMB3 | 1.4682 | [0.8608;2.0756] | <0.0001 |
| LAMC1 | 1.065 | [0.7405;1.3894] | <0.0001 |
| LAMC2 | 0.7921 | [0.2544;1.3297] | 0.0039 |
| LAMP2 | 0.8102 | [0.1755;1.4449] | 0.0124 |
| LAMP3 | 0.9901 | [0.2189;1.7614] | 0.0119 |
| LAPTM4B | 1.3833 | [0.7435;2.0230] | <0.0001 |
| LARGE | 0.5572 | [0.2194;0.8950] | 0.0012 |
| LARP6 | 1.2101 | [0.5311;1.8891] | 0.0005 |
| LCTL | 0.4331 | [0.0406;0.8257] | 0.0306 |
| LDHA | 0.8638 | [0.1491;1.5786] | 0.0179 |
| LDLRAD3 | 1.2348 | [0.8639;1.6057] | <0.0001 |
| LDOC1L | 1.4277 | [0.3638;2.4916] | 0.0085 |
| LEMD1 | 0.9846 | [0.1415;1.8278] | 0.0221 |
| LEPREL1 | 0.3595 | [0.0241;0.6949] | 0.0357 |
| LGALS1 | 1.256 | [0.9276;1.5844] | <0.0001 |
| LGALS3BP | 1.1188 | [0.6000;1.6377] | <0.0001 |
| LGALS7B | 0.7245 | [0.2319;1.2171] | 0.0039 |
| LGALS9 | 0.715 | [0.0694;1.3607] | 0.0299 |
| LGR4 | 1.247 | [0.5328;1.9611] | 0.0006 |
| LGR5 | 0.7687 | [0.4325;1.1049] | <0.0001 |
| LHFPL2 | 0.917 | [0.3031;1.5309] | 0.0034 |
| LHX2 | 1.6715 | [1.3281;2.0149] | <0.0001 |
| LIG1 | 0.9037 | [0.1474;1.6599] | 0.0192 |
| LIMK2 | 0.4176 | [0.1106;0.7246] | 0.0077 |
| LIN7C | 0.6773 | [0.1024;1.2523] | 0.0209 |
| LINC00277 | 0.4248 | [0.0047;0.8450] | 0.0475 |
| LITAF | 0.4398 | [0.1327;0.7469] | 0.005 |
| LLPH | 0.8507 | [0.5180;1.1834] | <0.0001 |
| LMAN1 | 1.1042 | [0.3987;1.8096] | 0.0022 |
| LMNB1 | 0.6876 | [0.3740;1.0012] | <0.0001 |
| LMNB2 | 1.6511 | [1.3008;2.0015] | <0.0001 |
| LMO4 | 0.5729 | [0.0033;1.1425] | 0.0487 |
| LNX2 | 0.5229 | [0.0042;1.0416] | 0.0482 |
| LOC284837 | 1.083 | [0.6828;1.4832] | <0.0001 |
| LOC344887 | 1.0727 | [0.2981;1.8472] | 0.0066 |
| LOC389834 | 0.992 | [0.0962;1.8877] | 0.03 |
| LONP1 | 0.666 | [0.0870;1.2450] | 0.0242 |
| LPAR6 | 0.7492 | [0.0378;1.4606] | 0.039 |
| LPCAT2 | 0.7017 | [0.1002;1.3031] | 0.0222 |
| LPHN1 | 0.9893 | [0.3214;1.6571] | 0.0037 |
| LPIN2 | 0.3845 | [0.0778;0.6912] | 0.014 |
| LPL | 0.6563 | [0.3466;0.9660] | <0.0001 |
| LRAT | 0.688 | [0.2932;1.0827] | 0.0006 |
| LRFN4 | 0.8268 | [0.5091;1.1445] | <0.0001 |
| LRIG3 | 1.5941 | [0.3969;2.7912] | 0.0091 |
| LRP1 | 0.5243 | [0.0232;1.0253] | 0.0403 |
| LRP12 | 1.0739 | [0.7352;1.4127] | <0.0001 |
| LRP4 | 0.8954 | [0.2280;1.5628] | 0.0086 |
| LRPPRC | 1.0452 | [0.7220;1.3684] | <0.0001 |
| LRR1 | 0.8653 | [0.1575;1.5730] | 0.0166 |
| LRRC1 | 1.0868 | [0.5196;1.6541] | 0.0002 |
| LRRC61 | 0.3709 | [0.0370;0.7047] | 0.0295 |
| LRRC8B | 0.4898 | [0.1579;0.8217] | 0.0038 |
| LRRCC1 | 1.0145 | [0.6489;1.3802] | <0.0001 |
| LSAMP | 0.5139 | [0.2077;0.8201] | 0.001 |
| LSM12 | 1.2515 | [0.4474;2.0556] | 0.0023 |
| LSM4 | 0.7726 | [0.4628;1.0825] | <0.0001 |
| LSM8 | 0.5492 | [0.1325;0.9660] | 0.0098 |
| LSR | 1.056 | [0.3055;1.8065] | 0.0058 |
| LTBR | 0.9736 | [0.2644;1.6828] | 0.0071 |
| LUZP1 | 0.6869 | [0.3729;1.0009] | <0.0001 |
| LY6E | 1.1855 | [0.5473;1.8238] | 0.0003 |
| LYPD6 | 0.808 | [0.1529;1.4631] | 0.0156 |
| LYSMD1 | 0.7347 | [0.0606;1.4088] | 0.0327 |
| LYZL6 | 0.3543 | [0.0384;0.6701] | 0.0279 |
| LZTS1 | 1.1802 | [0.4984;1.8620] | 0.0007 |
| MAD2L1 | 1.6463 | [1.0488;2.2439] | <0.0001 |
| MAGEB1 | 0.6021 | [0.2704;0.9339] | 0.0004 |
| MAGOH | 0.8914 | [0.4240;1.3587] | 0.0002 |
| MAGOHB | 1.3499 | [0.9817;1.7181] | <0.0001 |
| MAL2 | 1.4572 | [0.2295;2.6849] | 0.02 |
| MANEAL | 1.041 | [0.6754;1.4066] | <0.0001 |
| MAP7D2 | 0.9363 | [0.5861;1.2864] | <0.0001 |
| MAPK6 | 1.0111 | [0.4255;1.5967] | 0.0007 |
| MAPKBP1 | 1.1233 | [0.5353;1.7112] | 0.0002 |
| MARCO | 0.9535 | [0.6370;1.2700] | <0.0001 |
| MARK1 | 1.4384 | [0.8467;2.0302] | <0.0001 |
| MARVELD1 | 0.978 | [0.1595;1.7964] | 0.0192 |
| MARVELD3 | 1.3772 | [0.4392;2.3151] | 0.004 |
| MAST2 | 0.6654 | [0.0899;1.2409] | 0.0234 |
| MASTL | 1.1678 | [0.4752;1.8604] | 0.001 |
| MAT2A | 0.936 | [0.3644;1.5076] | 0.0013 |
| MB21D1 | 0.6497 | [0.2234;1.0760] | 0.0028 |
| MBNL2 | 0.5793 | [0.0868;1.0718] | 0.0211 |
| MCCC2 | 0.9055 | [0.5903;1.2206] | <0.0001 |
| MCFD2 | 0.3745 | [0.0696;0.6793] | 0.0161 |
| MCM10 | 1.3313 | [0.5463;2.1163] | 0.0009 |
| MCM2 | 1.1408 | [0.4611;1.8205] | 0.001 |
| MCM3 | 1.2777 | [0.3511;2.2043] | 0.0069 |
| MCM4 | 1.3452 | [0.6192;2.0711] | 0.0003 |
| MCM6 | 1.0887 | [0.2049;1.9725] | 0.0158 |
| MCM7 | 1.6064 | [0.6514;2.5615] | 0.001 |
| MCM8 | 1.0077 | [0.2261;1.7894] | 0.0115 |
| MCOLN3 | 0.3494 | [0.0459;0.6530] | 0.0241 |
| MDK | 1.5676 | [0.6338;2.5015] | 0.001 |
| MDM1 | 0.4632 | [0.1283;0.7980] | 0.0067 |
| MDM2 | 1.2681 | [0.9412;1.5950] | <0.0001 |
| MECOM | 0.7293 | [0.0894;1.3692] | 0.0255 |
| MEGF10 | 0.7184 | [0.3745;1.0622] | <0.0001 |
| MEGF8 | 0.5289 | [0.1924;0.8653] | 0.0021 |
| MELK | 1.3189 | [0.4560;2.1817] | 0.0027 |
| MERTK | 0.6927 | [0.3828;1.0025] | <0.0001 |
| MEST | 0.937 | [0.4215;1.4526] | 0.0004 |
| METTL8 | 0.7768 | [0.1677;1.3858] | 0.0124 |
| MEX3A | 1.2957 | [0.9191;1.6723] | <0.0001 |
| MEX3B | 0.5384 | [0.1925;0.8843] | 0.0023 |
| MEX3D | 0.8672 | [0.2334;1.5009] | 0.0073 |
| MFAP2 | 0.7521 | [0.4393;1.0649] | <0.0001 |
| MFAP3L | 0.491 | [0.0669;0.9152] | 0.0233 |
| MFHAS1 | 1.0075 | [0.6894;1.3256] | <0.0001 |
| MICAL2 | 1.3717 | [0.6540;2.0894] | 0.0002 |
| MICALL2 | 0.8323 | [0.5013;1.1634] | <0.0001 |
| MINPP1 | 1.1912 | [0.5961;1.7863] | <0.0001 |
| MIR22HG | 0.7893 | [0.2203;1.3584] | 0.0066 |
| MIS18A | 1.5556 | [1.1666;1.9446] | <0.0001 |
| MKI67 | 1.5171 | [0.4922;2.5419] | 0.0037 |
| MLF2 | 0.76 | [0.2351;1.2849] | 0.0045 |
| MLNR | 0.4031 | [0.0851;0.7211] | 0.013 |
| MMD | 0.7306 | [0.3918;1.0695] | <0.0001 |
| MMP1 | 1.5341 | [0.8916;2.1766] | <0.0001 |
| MMP11 | 0.8702 | [0.5498;1.1907] | <0.0001 |
| MMP12 | 0.8674 | [0.5487;1.1862] | <0.0001 |
| MMP13 | 0.3392 | [0.0329;0.6456] | 0.03 |
| MMP14 | 1.0229 | [0.3276;1.7181] | 0.0039 |
| MMP27 | 0.3681 | [0.0651;0.6710] | 0.0172 |
| MMP28 | 0.3189 | [0.0163;0.6216] | 0.0389 |
| MMP3 | 1.182 | [0.5620;1.8020] | 0.0002 |
| MMS22L | 1.3214 | [0.2491;2.3937] | 0.0157 |
| MN1 | 0.3971 | [0.0893;0.7048] | 0.0114 |
| MNX1 | 1.1512 | [0.8037;1.4987] | <0.0001 |
| MOCOS | 0.745 | [0.4311;1.0590] | <0.0001 |
| MORC2 | 0.769 | [0.0886;1.4493] | 0.0267 |
| MORC4 | 1.3649 | [0.7501;1.9798] | <0.0001 |
| MOV10 | 1.0544 | [0.7209;1.3880] | <0.0001 |
| MOV10L1 | 0.3271 | [0.0130;0.6412] | 0.0413 |
| MOXD1 | 0.7639 | [0.4526;1.0752] | <0.0001 |
| MPHOSPH6 | 0.5645 | [0.2523;0.8767] | 0.0004 |
| MPP2 | 0.4399 | [0.1339;0.7459] | 0.0048 |
| MPP3 | 1.5223 | [0.6748;2.3699] | 0.0004 |
| MPZL1 | 1.1799 | [0.8522;1.5076] | <0.0001 |
| MREG | 1.1402 | [0.4312;1.8492] | 0.0016 |
| MRGPRX3 | 0.3679 | [0.0145;0.7213] | 0.0413 |
| MRPL19 | 0.8934 | [0.3241;1.4627] | 0.0021 |
| MRPL3 | 1.2616 | [0.9311;1.5922] | <0.0001 |
| MRPL4 | 0.7327 | [0.1925;1.2729] | 0.0078 |
| MRPL42 | 1.2203 | [0.8898;1.5509] | <0.0001 |
| MRPL44 | 0.5717 | [0.0301;1.1134] | 0.0386 |
| MRPL47 | 1.3286 | [0.4447;2.2126] | 0.0032 |
| MRPL50 | 1.0208 | [0.3156;1.7260] | 0.0046 |
| MSANTD3 | 0.8715 | [0.3689;1.3741] | 0.0007 |
| MSH2 | 0.8905 | [0.5720;1.2090] | <0.0001 |
| MSH6 | 1.2745 | [0.7209;1.8281] | <0.0001 |
| MSR1 | 1.1564 | [0.8019;1.5109] | <0.0001 |
| MST1R | 0.4409 | [0.1334;0.7484] | 0.005 |
| MTBP | 0.9643 | [0.6282;1.3004] | <0.0001 |
| MTFR2 | 0.813 | [0.3091;1.3170] | 0.0016 |
| MTHFD2 | 0.8473 | [0.3349;1.3598] | 0.0012 |
| MTMR11 | 0.9788 | [0.3920;1.5656] | 0.0011 |
| MTMR4 | 0.8681 | [0.5511;1.1851] | <0.0001 |
| MTR | 0.8894 | [0.5754;1.2035] | <0.0001 |
| MTUS1 | 0.9316 | [0.2264;1.6367] | 0.0096 |
| MTX2 | 1.4204 | [1.0951;1.7458] | <0.0001 |
| MX1 | 0.6599 | [0.3488;0.9709] | <0.0001 |
| MYB | 0.8431 | [0.5326;1.1535] | <0.0001 |
| MYBPC1 | 0.5002 | [0.1979;0.8024] | 0.0012 |
| MYO10 | 0.9671 | [0.0859;1.8482] | 0.0315 |
| MYO19 | 1.4098 | [0.6934;2.1263] | 0.0001 |
| MYO1B | 1.2978 | [0.9714;1.6243] | <0.0001 |
| MYO3A | 0.7229 | [0.4071;1.0387] | <0.0001 |
| MYO3B | 0.9351 | [0.5825;1.2877] | <0.0001 |
| MYO6 | 1.0713 | [0.4129;1.7297] | 0.0014 |
| MYT1L | 0.4657 | [0.1481;0.7833] | 0.0041 |
| MZT1 | 1.0293 | [0.3557;1.7030] | 0.0027 |
| NAA15 | 0.9343 | [0.5944;1.2741] | <0.0001 |
| NAA25 | 1.0913 | [0.4052;1.7773] | 0.0018 |
| NANP | 1.1352 | [0.3570;1.9134] | 0.0042 |
| NARS | 0.9346 | [0.6065;1.2627] | <0.0001 |
| NAV1 | 0.9803 | [0.3545;1.6061] | 0.0021 |
| NAV2 | 1.2506 | [0.5323;1.9689] | 0.0006 |
| NCAPD3 | 0.8582 | [0.1083;1.6082] | 0.0249 |
| NCAPG | 1.2997 | [0.2614;2.3380] | 0.0142 |
| NCAPG2 | 1.0026 | [0.3670;1.6381] | 0.002 |
| NCBP2 | 0.8361 | [0.5202;1.1520] | <0.0001 |
| NCK1 | 1.3742 | [0.6045;2.1438] | 0.0005 |
| NCKAP1 | 0.8768 | [0.0309;1.7227] | 0.0422 |
| NCKAP5 | 0.685 | [0.3328;1.0371] | 0.0001 |
| NCS1 | 0.9175 | [0.1212;1.7139] | 0.0239 |
| NDC1 | 1.6535 | [1.1601;2.1469] | <0.0001 |
| NDC80 | 1.7227 | [0.5598;2.8857] | 0.0037 |
| NDRG1 | 0.3273 | [0.0232;0.6314] | 0.0349 |
| NDUFA5 | 0.7715 | [0.4331;1.1098] | <0.0001 |
| NDUFA6 | 0.9752 | [0.3209;1.6295] | 0.0035 |
| NDUFA9 | 0.9972 | [0.4140;1.5804] | 0.0008 |
| NECAB1 | 0.3643 | [0.0220;0.7066] | 0.037 |
| NEDD1 | 1.6235 | [1.2388;2.0083] | <0.0001 |
| NEIL3 | 0.8926 | [0.2064;1.5787] | 0.0108 |
| NEK2 | 1.3775 | [0.7704;1.9845] | <0.0001 |
| NET1 | 0.9566 | [0.1390;1.7742] | 0.0218 |
| NEURL1B | 0.7531 | [0.1569;1.3493] | 0.0133 |
| NEURL3 | 1.0094 | [0.6627;1.3560] | <0.0001 |
| NFE2L3 | 1.7015 | [0.9889;2.4142] | <0.0001 |
| NFKB2 | 0.3484 | [0.0186;0.6781] | 0.0384 |
| NFKBIA | 1.0081 | [0.6586;1.3575] | <0.0001 |
| NGFRAP1 | 1.1456 | [0.6117;1.6795] | <0.0001 |
| NHS | 1.4995 | [0.5902;2.4089] | 0.0012 |
| NHSL1 | 1.3775 | [0.4283;2.3268] | 0.0045 |
| NINJ1 | 0.7647 | [0.4481;1.0813] | <0.0001 |
| NIPSNAP1 | 1.4768 | [0.8338;2.1198] | <0.0001 |
| NIT2 | 1.2522 | [0.5924;1.9120] | 0.0002 |
| NKAIN1 | 0.3919 | [0.0645;0.7194] | 0.019 |
| NLN | 1.0168 | [0.3094;1.7243] | 0.0048 |
| NME1 | 1.3817 | [0.8510;1.9124] | <0.0001 |
| NME1-NME2 | 1.0445 | [0.2507;1.8383] | 0.0099 |
| NME2 | 0.8629 | [0.1846;1.5412] | 0.0127 |
| NME4 | 0.6772 | [0.3656;0.9888] | <0.0001 |
| NMI | 0.7881 | [0.1712;1.4050] | 0.0123 |
| NMNAT3 | 0.691 | [0.3418;1.0402] | 0.0001 |
| NNT | 0.6284 | [0.0994;1.1573] | 0.0199 |
| NOMO2 | 0.9305 | [0.2412;1.6199] | 0.0082 |
| NOP16 | 0.5577 | [0.2218;0.8937] | 0.0011 |
| NOP58 | 1.0137 | [0.0449;1.9826] | 0.0403 |
| NOTCH1 | 0.549 | [0.2133;0.8848] | 0.0014 |
| NOTCH3 | 0.7978 | [0.2176;1.3781] | 0.007 |
| NOTUM | 0.4299 | [0.0868;0.7730] | 0.0141 |
| NOV | 1.1854 | [0.8554;1.5155] | <0.0001 |
| NOX4 | 0.754 | [0.0233;1.4847] | 0.0431 |
| NPAS3 | 0.4849 | [0.1799;0.7899] | 0.0018 |
| NPL | 1.5324 | [1.1873;1.8775] | <0.0001 |
| NPNT | 0.8881 | [0.3064;1.4698] | 0.0028 |
| NPPC | 0.7104 | [0.4032;1.0176] | <0.0001 |
| NR2C2AP | 0.8717 | [0.5158;1.2276] | <0.0001 |
| NR2F6 | 0.7526 | [0.0392;1.4659] | 0.0387 |
| NR6A1 | 0.68 | [0.0662;1.2937] | 0.0299 |
| NRARP | 2.3758 | [0.3676;4.3839] | 0.0204 |
| NRCAM | 0.4325 | [0.1242;0.7408] | 0.006 |
| NRG1 | 0.7837 | [0.2560;1.3114] | 0.0036 |
| NRP1 | 0.4142 | [0.1054;0.7231] | 0.0086 |
| NRTN | 0.759 | [0.4509;1.0672] | <0.0001 |
| NRXN1 | 1.2143 | [0.8937;1.5349] | <0.0001 |
| NSDHL | 0.4155 | [0.1070;0.7241] | 0.0083 |
| NT5DC1 | 0.782 | [0.4293;1.1347] | <0.0001 |
| NT5M | 0.9684 | [0.3082;1.6285] | 0.004 |
| NTF3 | 0.7629 | [0.2476;1.2781] | 0.0037 |
| NTM | 0.8169 | [0.1517;1.4821] | 0.0161 |
| NTN1 | 0.874 | [0.1503;1.5978] | 0.0179 |
| NTRK2 | 1.1541 | [0.6094;1.6987] | <0.0001 |
| NUAK1 | 1.2114 | [0.8648;1.5579] | <0.0001 |
| NUB1 | 0.9256 | [0.5723;1.2789] | <0.0001 |
| NUCKS1 | 1.0554 | [0.4864;1.6244] | 0.0003 |
| NUDT1 | 0.7418 | [0.2082;1.2754] | 0.0064 |
| NUDT11 | 0.5885 | [0.2548;0.9222] | 0.0005 |
| NUDT12 | 0.8339 | [0.2279;1.4400] | 0.007 |
| NUDT15 | 1.0373 | [0.3623;1.7123] | 0.0026 |
| NUDT19 | 1.0506 | [0.3465;1.7547] | 0.0034 |
| NUF2 | 1.3818 | [0.4606;2.3030] | 0.0033 |
| NUP107 | 1.1146 | [0.3981;1.8311] | 0.0023 |
| NUP155 | 1.31 | [0.6961;1.9240] | <0.0001 |
| NUP35 | 0.9864 | [0.3539;1.6189] | 0.0022 |
| NUSAP1 | 1.3859 | [0.6182;2.1536] | 0.0004 |
| NXN | 0.9239 | [0.0094;1.8385] | 0.0477 |
| NXT2 | 0.8511 | [0.5355;1.1666] | <0.0001 |
| OAS1 | 0.6295 | [0.1176;1.1414] | 0.0159 |
| OAS2 | 0.7163 | [0.3800;1.0525] | <0.0001 |
| OAS3 | 1.119 | [0.7970;1.4411] | <0.0001 |
| OASL | 1.0919 | [0.5198;1.6639] | 0.0002 |
| ODF3L1 | 0.4504 | [0.1106;0.7903] | 0.0094 |
| OGFRL1 | 0.7017 | [0.4004;1.0031] | <0.0001 |
| OIP5 | 1.2194 | [0.3282;2.1106] | 0.0073 |
| OLA1 | 1.3908 | [1.0370;1.7445] | <0.0001 |
| OLFM1 | 0.8569 | [0.5419;1.1719] | <0.0001 |
| OLFM3 | 0.3793 | [0.0509;0.7077] | 0.0236 |
| OLFM4 | 0.5854 | [0.2795;0.8914] | 0.0002 |
| OLR1 | 1.2247 | [0.6945;1.7550] | <0.0001 |
| ONECUT2 | 0.6416 | [0.0194;1.2638] | 0.0433 |
| OR5P2 | 0.7181 | [0.3781;1.0582] | <0.0001 |
| OR5P3 | 0.3998 | [0.0677;0.7319] | 0.0183 |
| ORC6 | 1.3437 | [0.4737;2.2138] | 0.0025 |
| OSBPL1A | 0.9337 | [0.2335;1.6338] | 0.009 |
| OSM | 0.4981 | [0.1939;0.8023] | 0.0013 |
| OTULIN | 0.9431 | [0.4041;1.4820] | 0.0006 |
| P2RX4 | 0.4113 | [0.1037;0.7188] | 0.0088 |
| P4HA1 | 1.0589 | [0.7387;1.3791] | <0.0001 |
| P4HA2 | 0.7299 | [0.0877;1.3720] | 0.0259 |
| PACRGL | 0.931 | [0.3201;1.5419] | 0.0028 |
| PACSIN3 | 0.8289 | [0.1263;1.5315] | 0.0208 |
| PADI3 | 0.4038 | [0.0953;0.7122] | 0.0103 |
| PAFAH1B2 | 0.4589 | [0.1297;0.7881] | 0.0063 |
| PAICS | 1.2922 | [0.9549;1.6295] | <0.0001 |
| PAIP1 | 1.2482 | [0.6168;1.8796] | 0.0001 |
| PAK1IP1 | 0.9069 | [0.3088;1.5050] | 0.003 |
| PAK3 | 0.5738 | [0.0249;1.1226] | 0.0405 |
| PAK4 | 0.8318 | [0.0466;1.6170] | 0.0379 |
| PALB2 | 1.2668 | [0.5793;1.9542] | 0.0003 |
| PAMR1 | 0.3867 | [0.0570;0.7164] | 0.0215 |
| PANK1 | 0.7302 | [0.4058;1.0546] | <0.0001 |
| PANX1 | 0.9101 | [0.5905;1.2297] | <0.0001 |
| PAPSS2 | 1.1205 | [0.7391;1.5020] | <0.0001 |
| PAQR3 | 0.4272 | [0.1241;0.7304] | 0.0057 |
| PAQR4 | 0.8041 | [0.1356;1.4726] | 0.0184 |
| PAQR5 | 0.6869 | [0.3486;1.0252] | <0.0001 |
| PAQR6 | 0.7553 | [0.1034;1.4072] | 0.0232 |
| PARD3B | 1.0877 | [0.4756;1.6998] | 0.0005 |
| PARD6B | 0.6303 | [0.0606;1.2000] | 0.0301 |
| PARD6G | 1.0292 | [0.3215;1.7368] | 0.0044 |
| PARM1 | 0.769 | [0.4299;1.1082] | <0.0001 |
| PARP12 | 1.0779 | [0.3619;1.7939] | 0.0032 |
| PARP14 | 0.9535 | [0.1042;1.8028] | 0.0278 |
| PARP9 | 1.1914 | [0.3827;2.0001] | 0.0039 |
| PARPBP | 1.9646 | [1.4353;2.4939] | <0.0001 |
| PAX6 | 0.5313 | [0.2249;0.8376] | 0.0007 |
| PBK | 1.5085 | [1.1334;1.8835] | <0.0001 |
| PBX1 | 1.3012 | [0.5227;2.0796] | 0.0011 |
| PCDH19 | 0.7532 | [0.4059;1.1005] | <0.0001 |
| PCDHB6 | 0.3132 | [0.0109;0.6156] | 0.0423 |
| PCDHB8 | 0.3369 | [0.0298;0.6441] | 0.0316 |
| PCDHB9 | 0.628 | [0.2746;0.9813] | 0.0005 |
| PCGF3 | 0.4072 | [0.0825;0.7320] | 0.014 |
| PCNA | 1.2794 | [0.4829;2.0760] | 0.0016 |
| PCOLCE2 | 1.0219 | [0.6988;1.3451] | <0.0001 |
| PCSK6 | 1.2226 | [0.5507;1.8945] | 0.0004 |
| PCSK9 | 0.6341 | [0.2899;0.9783] | 0.0003 |
| PDCD1LG2 | 0.376 | [0.0674;0.6847] | 0.017 |
| PDCD2L | 1.0501 | [0.0967;2.0035] | 0.0309 |
| PDCD5 | 1.6173 | [1.2698;1.9649] | <0.0001 |
| PDCL | 0.6017 | [0.2618;0.9416] | 0.0005 |
| PDGFA | 0.5997 | [0.0293;1.1702] | 0.0394 |
| PDGFC | 0.3332 | [0.0285;0.6379] | 0.0321 |
| PDGFRL | 0.8478 | [0.5356;1.1600] | <0.0001 |
| PELI1 | 1.0736 | [0.7527;1.3945] | <0.0001 |
| PERP | 1.3937 | [0.2755;2.5119] | 0.0146 |
| PFDN2 | 0.6598 | [0.0026;1.3170] | 0.0491 |
| PFDN4 | 1.3348 | [1.0035;1.6660] | <0.0001 |
| PGAM5 | 1.1395 | [0.3785;1.9006] | 0.0033 |
| PGAP1 | 1.6992 | [1.3314;2.0670] | <0.0001 |
| PGLYRP3 | 0.9677 | [0.0577;1.8777] | 0.0371 |
| PGLYRP4 | 1.1634 | [0.4575;1.8693] | 0.0012 |
| PGM2 | 1.0448 | [0.3625;1.7271] | 0.0027 |
| PGRMC1 | 1.3425 | [0.7353;1.9498] | <0.0001 |
| PHF14 | 1.1823 | [0.5331;1.8316] | 0.0004 |
| PHF21B | 0.9362 | [0.2798;1.5927] | 0.0052 |
| PHGDH | 1.0249 | [0.6797;1.3701] | <0.0001 |
| PHKA1 | 0.6567 | [0.3421;0.9714] | <0.0001 |
| PHLDA1 | 0.7245 | [0.0959;1.3531] | 0.0239 |
| PHLDA3 | 1.3184 | [0.5576;2.0792] | 0.0007 |
| PHOSPHO2 | 0.5682 | [0.1910;0.9453] | 0.0032 |
| PIGW | 1.2127 | [0.4681;1.9573] | 0.0014 |
| PIK3CB | 1.4361 | [0.7719;2.1002] | <0.0001 |
| PIK3R2 | 0.8559 | [0.0939;1.6179] | 0.0277 |
| PIK3R3 | 0.7664 | [0.1019;1.4309] | 0.0238 |
| PIM3 | 1.1271 | [0.3376;1.9165] | 0.0051 |
| PIR | 0.9191 | [0.2627;1.5754] | 0.0061 |
| PKDCC | 1.0104 | [0.3180;1.7028] | 0.0042 |
| PKP1 | 1.4508 | [0.5863;2.3153] | 0.001 |
| PKP3 | 1.4218 | [0.2869;2.5567] | 0.0141 |
| PKP4 | 1.2859 | [0.4445;2.1272] | 0.0027 |
| PLA2G3 | 1.3836 | [0.7588;2.0083] | <0.0001 |
| PLA2G4A | 0.6478 | [0.3357;0.9599] | <0.0001 |
| PLA2G7 | 0.6533 | [0.0171;1.2895] | 0.0442 |
| PLAGL2 | 1.0205 | [0.4235;1.6176] | 0.0008 |
| PLAU | 1.8921 | [1.1723;2.6119] | <0.0001 |
| PLAUR | 1.1066 | [0.7832;1.4300] | <0.0001 |
| PLCB1 | 0.6601 | [0.3523;0.9679] | <0.0001 |
| PLCB4 | 0.3594 | [0.0543;0.6644] | 0.0209 |
| PLEK2 | 1.5494 | [0.6939;2.4049] | 0.0004 |
| PLEKHA1 | 1.0593 | [0.2685;1.8501] | 0.0087 |
| PLEKHA5 | 0.7436 | [0.0662;1.4211] | 0.0314 |
| PLEKHG3 | 1.1034 | [0.2040;2.0027] | 0.0162 |
| PLK4 | 1.1694 | [0.5023;1.8366] | 0.0006 |
| PLOD1 | 0.8311 | [0.2542;1.4079] | 0.0047 |
| PLOD2 | 0.5862 | [0.0563;1.1161] | 0.0301 |
| PLS3 | 1.1923 | [0.4962;1.8883] | 0.0008 |
| PLSCR1 | 0.7751 | [0.4622;1.0880] | <0.0001 |
| PLXDC2 | 1.0353 | [0.7104;1.3602] | <0.0001 |
| PLXNA1 | 1.6884 | [0.8472;2.5295] | <0.0001 |
| PLXNA2 | 0.8528 | [0.1221;1.5836] | 0.0222 |
| PLXNB1 | 0.6046 | [0.0249;1.1843] | 0.0409 |
| PM20D2 | 0.8454 | [0.4911;1.1996] | <0.0001 |
| PMAIP1 | 1.6188 | [1.2471;1.9905] | <0.0001 |
| PMCH | 0.5092 | [0.2048;0.8137] | 0.001 |
| PMEPA1 | 0.4222 | [0.0935;0.7508] | 0.0118 |
| PML | 0.7577 | [0.2208;1.2945] | 0.0057 |
| PMS1 | 0.8489 | [0.3277;1.3701] | 0.0014 |
| PNLIPRP1 | 0.3369 | [0.0101;0.6636] | 0.0433 |
| PNPT1 | 1.089 | [0.7549;1.4231] | <0.0001 |
| PNRC1 | 0.509 | [0.2043;0.8137] | 0.0011 |
| PODXL | 0.8003 | [0.4862;1.1143] | <0.0001 |
| POGLUT1 | 1.2154 | [0.8710;1.5598] | <0.0001 |
| POLB | 0.956 | [0.6400;1.2720] | <0.0001 |
| POLD2 | 0.9033 | [0.3909;1.4157] | 0.0005 |
| POLDIP2 | 0.8225 | [0.2935;1.3514] | 0.0023 |
| POLE | 0.84 | [0.0093;1.6706] | 0.0475 |
| POLE2 | 0.8405 | [0.2130;1.4680] | 0.0087 |
| POLQ | 1.0359 | [0.2446;1.8272] | 0.0103 |
| POLR1A | 0.8105 | [0.1575;1.4635] | 0.015 |
| PON2 | 0.8332 | [0.5241;1.1423] | <0.0001 |
| POP1 | 0.7071 | [0.0210;1.3931] | 0.0434 |
| POPDC3 | 0.3682 | [0.0377;0.6987] | 0.029 |
| POSTN | 1.0902 | [0.7677;1.4127] | <0.0001 |
| PPA1 | 1.1708 | [0.8174;1.5241] | <0.0001 |
| PPAPDC1A | 1.0835 | [0.3333;1.8338] | 0.0046 |
| PPARGC1B | 1.2406 | [0.5017;1.9795] | 0.001 |
| PPEF1 | 0.5264 | [0.2184;0.8344] | 0.0008 |
| PPFIA1 | 0.9731 | [0.6329;1.3133] | <0.0001 |
| PPFIA4 | 0.701 | [0.1720;1.2299] | 0.0094 |
| PPFIBP1 | 0.5533 | [0.2472;0.8594] | 0.0004 |
| PPFIBP2 | 0.5315 | [0.0275;1.0355] | 0.0387 |
| PPIA | 0.9449 | [0.3527;1.5371] | 0.0018 |
| PPIF | 1.0441 | [0.4970;1.5912] | 0.0002 |
| PPIL1 | 0.406 | [0.0591;0.7528] | 0.0218 |
| PPP1R14C | 0.7164 | [0.0048;1.4279] | 0.0485 |
| PPP1R15B | 0.9278 | [0.3142;1.5414] | 0.003 |
| PPP2R2C | 0.9491 | [0.1595;1.7388] | 0.0185 |
| PPP4R1 | 0.7753 | [0.1038;1.4468] | 0.0236 |
| PPP4R4 | 0.8139 | [0.4702;1.1576] | <0.0001 |
| PPTC7 | 0.9039 | [0.2427;1.5651] | 0.0074 |
| PRC1 | 1.6134 | [0.8360;2.3908] | <0.0001 |
| PRCC | 0.6494 | [0.3333;0.9654] | <0.0001 |
| PRG3 | 0.3368 | [0.0084;0.6651] | 0.0444 |
| PRIM2 | 0.8935 | [0.2197;1.5674] | 0.0094 |
| PRIMA1 | 0.6298 | [0.2884;0.9712] | 0.0003 |
| PRKAR2B | 0.5572 | [0.2485;0.8660] | 0.0004 |
| PRKCI | 0.9866 | [0.3691;1.6040] | 0.0017 |
| PRKDC | 1.2093 | [0.8790;1.5397] | <0.0001 |
| PRLR | 1.013 | [0.6925;1.3334] | <0.0001 |
| PRNP | 1.1576 | [0.6241;1.6911] | <0.0001 |
| PRODH | 1.4242 | [0.2938;2.5546] | 0.0135 |
| PROX1 | 1.0701 | [0.7232;1.4171] | <0.0001 |
| PRR5L | 1.1161 | [0.7683;1.4638] | <0.0001 |
| PRRX1 | 1.19 | [0.8683;1.5117] | <0.0001 |
| PRSS8 | 1.1328 | [0.0694;2.1962] | 0.0368 |
| PRTG | 0.5607 | [0.2197;0.9018] | 0.0013 |
| PSAT1 | 1.4363 | [1.0697;1.8029] | <0.0001 |
| PSD3 | 1.058 | [0.4690;1.6470] | 0.0004 |
| PSMA2 | 1.0854 | [0.7608;1.4100] | <0.0001 |
| PSMA3 | 0.5667 | [0.0500;1.0835] | 0.0316 |
| PSMA4 | 1.4051 | [1.0628;1.7474] | <0.0001 |
| PSMB4 | 0.8638 | [0.3018;1.4257] | 0.0026 |
| PSMC3IP | 1.6251 | [0.9317;2.3185] | <0.0001 |
| PSMD14 | 1.2607 | [0.7029;1.8184] | <0.0001 |
| PSMD9 | 0.7055 | [0.3891;1.0218] | <0.0001 |
| PSPH | 1.2369 | [0.6508;1.8229] | <0.0001 |
| PSRC1 | 1.0046 | [0.2031;1.8061] | 0.014 |
| PSTPIP2 | 0.9178 | [0.6025;1.2330] | <0.0001 |
| PTCH1 | 0.5279 | [0.1987;0.8571] | 0.0017 |
| PTGES | 0.6519 | [0.0885;1.2153] | 0.0233 |
| PTGFRN | 1.3839 | [0.3794;2.3883] | 0.0069 |
| PTGS2 | 1.5416 | [1.2057;1.8775] | <0.0001 |
| PTH2R | 0.3372 | [0.0160;0.6584] | 0.0397 |
| PTK7 | 1.1656 | [0.3655;1.9656] | 0.0043 |
| PTMS | 0.688 | [0.0679;1.3081] | 0.0297 |
| PTPN13 | 1.0579 | [0.4627;1.6532] | 0.0005 |
| PTPN14 | 1.2101 | [0.6788;1.7414] | <0.0001 |
| PTPRF | 1.7188 | [0.5974;2.8402] | 0.0027 |
| PTPRG | 0.5305 | [0.1946;0.8663] | 0.002 |
| PTPRK | 1.1994 | [0.4088;1.9900] | 0.0029 |
| PTPRR | 0.8763 | [0.5625;1.1900] | <0.0001 |
| PTTG1 | 1.313 | [0.5709;2.0551] | 0.0005 |
| PTTG3P | 0.7137 | [0.0401;1.3874] | 0.0378 |
| PUS7 | 1.7052 | [1.3224;2.0879] | <0.0001 |
| PVR | 0.9176 | [0.1968;1.6383] | 0.0126 |
| PXDN | 0.7685 | [0.4284;1.1086] | <0.0001 |
| PXYLP1 | 0.7791 | [0.2739;1.2843] | 0.0025 |
| PYCARD | 0.8816 | [0.1621;1.6012] | 0.0163 |
| PYCR1 | 1.0091 | [0.3968;1.6213] | 0.0012 |
| QKI | 0.7052 | [0.2087;1.2017] | 0.0054 |
| QSER1 | 1.3238 | [0.5525;2.0951] | 0.0008 |
| RAB13 | 1.0536 | [0.3631;1.7440] | 0.0028 |
| RAB23 | 0.7223 | [0.4065;1.0381] | <0.0001 |
| RAB25 | 1.0276 | [0.0839;1.9713] | 0.0328 |
| RAB27B | 0.7469 | [0.0714;1.4225] | 0.0302 |
| RAB34 | 0.7457 | [0.3659;1.1254] | 0.0001 |
| RAB9A | 1.2 | [0.5547;1.8453] | 0.0003 |
| RACGAP1 | 1.3384 | [0.6536;2.0232] | 0.0001 |
| RAD51 | 0.8886 | [0.3006;1.4766] | 0.0031 |
| RAD51AP1 | 2.0004 | [1.2023;2.7985] | <0.0001 |
| RAD54B | 1.2698 | [0.9326;1.6070] | <0.0001 |
| RAD54L | 1.0044 | [0.2682;1.7405] | 0.0075 |
| RAD9B | 0.5146 | [0.1964;0.8327] | 0.0015 |
| RAET1K | 1.2113 | [0.6543;1.7683] | <0.0001 |
| RAET1L | 1.3016 | [0.4293;2.1738] | 0.0035 |
| RAI14 | 1.0471 | [0.5448;1.5494] | <0.0001 |
| RALA | 1.4555 | [0.7651;2.1459] | <0.0001 |
| RAMP1 | 0.6145 | [0.3115;0.9176] | <0.0001 |
| RAN | 1.1679 | [0.5206;1.8152] | 0.0004 |
| RANBP1 | 0.7827 | [0.4628;1.1026] | <0.0001 |
| RANBP17 | 0.9837 | [0.6409;1.3264] | <0.0001 |
| RAP2A | 0.6191 | [0.3128;0.9254] | <0.0001 |
| RAPGEFL1 | 1.0737 | [0.3363;1.8110] | 0.0043 |
| RARG | 0.8355 | [0.0197;1.6513] | 0.0447 |
| RASGEF1B | 0.795 | [0.0930;1.4970] | 0.0265 |
| RASSF10 | 0.6483 | [0.0015;1.2950] | 0.0495 |
| RASSF4 | 0.5207 | [0.2108;0.8305] | 0.001 |
| RASSF8 | 0.6817 | [0.3430;1.0204] | <0.0001 |
| RAVER2 | 0.6249 | [0.2893;0.9606] | 0.0003 |
| RBBP8 | 1.6145 | [0.8340;2.3949] | <0.0001 |
| RBFOX2 | 1.1458 | [0.4268;1.8648] | 0.0018 |
| RBL1 | 0.6844 | [0.0027;1.3661] | 0.0491 |
| RCC1 | 0.879 | [0.5376;1.2205] | <0.0001 |
| RCC2 | 1.048 | [0.2712;1.8249] | 0.0082 |
| RCHY1 | 0.3911 | [0.0839;0.6982] | 0.0126 |
| RCN1 | 2.3258 | [1.6211;3.0305] | <0.0001 |
| RCN2 | 1.7194 | [1.3768;2.0621] | <0.0001 |
| RDM1 | 0.9711 | [0.2964;1.6458] | 0.0048 |
| RELB | 0.7622 | [0.4235;1.1009] | <0.0001 |
| REPIN1 | 0.7814 | [0.1578;1.4049] | 0.0141 |
| RET | 0.5253 | [0.1955;0.8551] | 0.0018 |
| RFC2 | 0.9443 | [0.3521;1.5366] | 0.0018 |
| RFC3 | 1.0987 | [0.3748;1.8226] | 0.0029 |
| RFC4 | 1.1367 | [0.4282;1.8453] | 0.0017 |
| RFC5 | 1.5299 | [0.7549;2.3049] | 0.0001 |
| RFX4 | 0.4282 | [0.1262;0.7302] | 0.0055 |
| RFX5 | 0.6026 | [0.0543;1.1509] | 0.0312 |
| RGAG1 | 0.444 | [0.0984;0.7895] | 0.0118 |
| RGPD6 | 0.9383 | [0.0657;1.8109] | 0.0351 |
| RGS1 | 0.6635 | [0.3325;0.9946] | <0.0001 |
| RGS12 | 1.05 | [0.2860;1.8140] | 0.0071 |
| RGS16 | 0.5419 | [0.2315;0.8523] | 0.0006 |
| RGS20 | 0.5741 | [0.0613;1.0869] | 0.0282 |
| RGS4 | 0.6238 | [0.2922;0.9555] | 0.0002 |
| RHBDL3 | 0.76 | [0.4112;1.1088] | <0.0001 |
| RHEB | 1.3824 | [0.8654;1.8993] | <0.0001 |
| RHOBTB3 | 0.7123 | [0.3984;1.0262] | <0.0001 |
| RHOT1 | 1.1887 | [0.8648;1.5127] | <0.0001 |
| RIF1 | 1.5234 | [0.5508;2.4959] | 0.0021 |
| RILPL1 | 0.7193 | [0.3642;1.0745] | <0.0001 |
| RIN1 | 0.9293 | [0.2426;1.6159] | 0.008 |
| RIPK4 | 1.1306 | [0.0879;2.1733] | 0.0336 |
| RMI1 | 0.7452 | [0.4003;1.0902] | <0.0001 |
| RMI2 | 1.2749 | [0.8762;1.6737] | <0.0001 |
| RNASE2 | 0.4706 | [0.1673;0.7738] | 0.0024 |
| RNASEH2A | 0.9249 | [0.3539;1.4958] | 0.0015 |
| RNF114 | 0.9772 | [0.3270;1.6274] | 0.0032 |
| RNF148 | 0.9632 | [0.2210;1.7054] | 0.011 |
| RNF165 | 1.5817 | [0.8347;2.3288] | <0.0001 |
| RNF19B | 1.1252 | [0.5742;1.6762] | <0.0001 |
| RNF43 | 1.7669 | [0.9489;2.5849] | <0.0001 |
| ROBO1 | 1.4458 | [1.1144;1.7772] | <0.0001 |
| RPF2 | 0.9897 | [0.6410;1.3385] | <0.0001 |
| RPL22L1 | 0.7082 | [0.3294;1.0870] | 0.0002 |
| RPS6KA6 | 0.3806 | [0.0773;0.6839] | 0.0139 |
| RRM1 | 1.1078 | [0.7806;1.4350] | <0.0001 |
| RRM2 | 1.3877 | [0.6586;2.1167] | 0.0002 |
| RSAD2 | 1.2963 | [0.9449;1.6477] | <0.0001 |
| RSRC1 | 1.2765 | [0.6407;1.9123] | <0.0001 |
| RTKN | 1.3687 | [0.7155;2.0219] | <0.0001 |
| RTKN2 | 0.7155 | [0.3661;1.0648] | <0.0001 |
| RTN4RL1 | 1.3508 | [0.6730;2.0285] | <0.0001 |
| RTP3 | 0.4891 | [0.1242;0.8539] | 0.0086 |
| RWDD2B | 0.7221 | [0.3813;1.0629] | <0.0001 |
| RXRA | 0.9069 | [0.2092;1.6047] | 0.0108 |
| S100A11 | 0.8383 | [0.0370;1.6395] | 0.0403 |
| S100A14 | 1.0599 | [0.1670;1.9529] | 0.02 |
| S1PR5 | 0.9356 | [0.0598;1.8113] | 0.0363 |
| SAC3D1 | 1.1007 | [0.4554;1.7461] | 0.0008 |
| SALL1 | 0.3828 | [0.0823;0.6832] | 0.0125 |
| SAMD1 | 0.9613 | [0.2187;1.7038] | 0.0112 |
| SAMD12 | 1.3527 | [0.6235;2.0820] | 0.0003 |
| SAMD9L | 0.7456 | [0.0888;1.4023] | 0.0261 |
| SASS6 | 0.9552 | [0.1818;1.7286] | 0.0155 |
| SCAI | 0.8025 | [0.1663;1.4387] | 0.0134 |
| SCAMP1 | 0.4157 | [0.1087;0.7227] | 0.008 |
| SCARB2 | 1.0772 | [0.3345;1.8200] | 0.0045 |
| SCD | 1.3422 | [0.5899;2.0944] | 0.0005 |
| SCD5 | 1.5046 | [0.7433;2.2658] | 0.0001 |
| SCLY | 1.1141 | [0.7592;1.4690] | <0.0001 |
| SCML2 | 1.0118 | [0.6905;1.3331] | <0.0001 |
| SCN10A | 0.4046 | [0.0853;0.7239] | 0.013 |
| SCNN1G | 1.1775 | [0.8278;1.5271] | <0.0001 |
| SCO2 | 0.7586 | [0.2509;1.2663] | 0.0034 |
| SCOC | 0.5358 | [0.2211;0.8505] | 0.0008 |
| SCPEP1 | 0.7162 | [0.2010;1.2315] | 0.0064 |
| SCRN3 | 0.5248 | [0.2158;0.8338] | 0.0009 |
| SCUBE3 | 0.4568 | [0.1285;0.7851] | 0.0064 |
| SDC2 | 0.8366 | [0.5219;1.1513] | <0.0001 |
| SDC4 | 1.2511 | [0.2748;2.2273] | 0.012 |
| SDK2 | 0.797 | [0.1451;1.4488] | 0.0166 |
| SDR9C7 | 0.7534 | [0.4046;1.1021] | <0.0001 |
| SDS | 0.8349 | [0.2341;1.4357] | 0.0065 |
| SEC61A2 | 1.0892 | [0.5640;1.6144] | <0.0001 |
| SEMA4C | 1.0527 | [0.4813;1.6240] | 0.0003 |
| SEMA6A | 0.7912 | [0.2667;1.3157] | 0.0031 |
| SERF1A | 1.2401 | [0.7696;1.7106] | <0.0001 |
| SERPINE1 | 0.9229 | [0.4164;1.4294] | 0.0004 |
| SERPINE2 | 0.3847 | [0.0563;0.7131] | 0.0217 |
| SERPINH1 | 0.8679 | [0.5495;1.1863] | <0.0001 |
| SFRP1 | 0.475 | [0.1683;0.7817] | 0.0024 |
| SFXN1 | 0.8453 | [0.2612;1.4294] | 0.0046 |
| SGIP1 | 0.6274 | [0.0223;1.2325] | 0.0421 |
| SGK1 | 1.8008 | [0.9018;2.6997] | <0.0001 |
| SGPP2 | 1.6818 | [0.4914;2.8722] | 0.0056 |
| SH2D4A | 1.1267 | [0.1878;2.0656] | 0.0187 |
| SH3BGR | 0.5077 | [0.1996;0.8158] | 0.0012 |
| SH3PXD2A | 0.6788 | [0.3431;1.0146] | <0.0001 |
| SH3YL1 | 0.8016 | [0.4878;1.1155] | <0.0001 |
| SHB | 0.7523 | [0.2553;1.2493] | 0.003 |
| SHC3 | 0.6631 | [0.1315;1.1946] | 0.0145 |
| SHFM1 | 0.9042 | [0.2943;1.5140] | 0.0037 |
| SHISA2 | 0.6019 | [0.2611;0.9428] | 0.0005 |
| SHOX2 | 0.6707 | [0.3375;1.0039] | <0.0001 |
| SHROOM2 | 1.3901 | [0.6234;2.1568] | 0.0004 |
| SHROOM3 | 1.2205 | [0.0163;2.4246] | 0.047 |
| SIGLEC1 | 0.7343 | [0.3991;1.0696] | <0.0001 |
| SIK1 | 1.0918 | [0.1791;2.0045] | 0.0191 |
| SIM2 | 0.6192 | [0.0069;1.2316] | 0.0475 |
| SIMC1 | 0.4755 | [0.0228;0.9283] | 0.0395 |
| SIPA1L2 | 0.7195 | [0.1830;1.2560] | 0.0086 |
| SIPA1L3 | 0.575 | [0.0470;1.1030] | 0.0328 |
| SIRPA | 0.3334 | [0.0044;0.6623] | 0.047 |
| SKA1 | 0.9476 | [0.0924;1.8028] | 0.0299 |
| SKA2 | 0.6803 | [0.0551;1.3055] | 0.033 |
| SKP2 | 1.2456 | [0.6047;1.8864] | 0.0001 |
| SLBP | 0.9549 | [0.6328;1.2770] | <0.0001 |
| SLC11A1 | 0.6151 | [0.1279;1.1022] | 0.0133 |
| SLC12A7 | 0.8492 | [0.5114;1.1869] | <0.0001 |
| SLC16A1 | 0.761 | [0.2371;1.2849] | 0.0044 |
| SLC1A3 | 0.8304 | [0.5132;1.1476] | <0.0001 |
| SLC25A10 | 0.7698 | [0.1719;1.3676] | 0.0116 |
| SLC25A13 | 1.078 | [0.4599;1.6962] | 0.0006 |
| SLC25A21 | 0.4968 | [0.1683;0.8253] | 0.003 |
| SLC25A22 | 0.6938 | [0.3788;1.0089] | <0.0001 |
| SLC25A33 | 0.759 | [0.4047;1.1133] | <0.0001 |
| SLC25A40 | 0.9928 | [0.6180;1.3676] | <0.0001 |
| SLC26A10 | 0.3356 | [0.0310;0.6402] | 0.0308 |
| SLC28A3 | 1.2636 | [0.7400;1.7871] | <0.0001 |
| SLC29A4 | 0.6386 | [0.0042;1.2731] | 0.0485 |
| SLC2A1 | 0.6286 | [0.1190;1.1382] | 0.0156 |
| SLC2A12 | 0.4088 | [0.0963;0.7212] | 0.0103 |
| SLC2A13 | 0.8315 | [0.5060;1.1569] | <0.0001 |
| SLC30A6 | 0.782 | [0.4540;1.1100] | <0.0001 |
| SLC35F2 | 0.8794 | [0.2119;1.5469] | 0.0098 |
| SLC36A1 | 0.995 | [0.2554;1.7345] | 0.0084 |
| SLC37A3 | 0.886 | [0.2995;1.4726] | 0.0031 |
| SLC39A11 | 1.3916 | [0.7945;1.9887] | <0.0001 |
| SLC39A14 | 0.7867 | [0.1817;1.3917] | 0.0108 |
| SLC41A2 | 0.4786 | [0.1617;0.7956] | 0.0031 |
| SLC44A1 | 1.2997 | [0.4838;2.1156] | 0.0018 |
| SLC44A5 | 0.4585 | [0.1119;0.8050] | 0.0095 |
| SLC5A6 | 1.5231 | [0.7980;2.2481] | <0.0001 |
| SLC6A15 | 0.4148 | [0.1084;0.7212] | 0.008 |
| SLC7A5 | 0.6064 | [0.0999;1.1130] | 0.019 |
| SLC9A7 | 0.9952 | [0.4345;1.5560] | 0.0005 |
| SLCO1A2 | 0.7589 | [0.2251;1.2927] | 0.0053 |
| SLCO5A1 | 1.0082 | [0.3563;1.6602] | 0.0024 |
| SLIRP | 0.8212 | [0.1702;1.4722] | 0.0134 |
| SMAD1 | 1.1306 | [0.5979;1.6634] | <0.0001 |
| SMAD4 | 0.3778 | [0.0728;0.6828] | 0.0152 |
| SMAD5-AS1 | 0.623 | [0.2465;0.9995] | 0.0012 |
| SMARCA4 | 1.3337 | [0.6030;2.0644] | 0.0003 |
| SMARCAD1 | 1.1376 | [0.4264;1.8489] | 0.0017 |
| SMC2 | 1.0827 | [0.2570;1.9083] | 0.0102 |
| SMC4 | 1.6125 | [0.8537;2.3713] | <0.0001 |
| SMURF2 | 0.3813 | [0.0776;0.6851] | 0.0139 |
| SMYD2 | 0.446 | [0.1428;0.7493] | 0.0039 |
| SNAI2 | 0.9351 | [0.3674;1.5027] | 0.0012 |
| SNORA33 | 1.256 | [0.4862;2.0259] | 0.0014 |
| SNRPD1 | 1.0253 | [0.3420;1.7086] | 0.0033 |
| SNRPE | 0.9982 | [0.6744;1.3220] | <0.0001 |
| SNX10 | 0.8051 | [0.2903;1.3200] | 0.0022 |
| SNX24 | 0.4894 | [0.1793;0.7995] | 0.002 |
| SNX4 | 0.8585 | [0.5475;1.1695] | <0.0001 |
| SOCS1 | 1.0165 | [0.6767;1.3563] | <0.0001 |
| SOCS2 | 0.5727 | [0.2656;0.8798] | 0.0003 |
| SOCS5 | 0.8705 | [0.5582;1.1828] | <0.0001 |
| SOD1 | 0.5606 | [0.2478;0.8733] | 0.0004 |
| SORD | 1.4342 | [0.7228;2.1457] | <0.0001 |
| SOST | 0.5601 | [0.0583;1.0619] | 0.0287 |
| SOX11 | 0.6674 | [0.3574;0.9774] | <0.0001 |
| SOX13 | 0.7621 | [0.1147;1.4094] | 0.021 |
| SOX2 | 1.2418 | [0.3083;2.1753] | 0.0091 |
| SOX4 | 2.0477 | [1.1670;2.9283] | <0.0001 |
| SOX9 | 1.0023 | [0.3378;1.6667] | 0.0031 |
| SP6 | 0.8346 | [0.4276;1.2416] | <0.0001 |
| SPAG5 | 0.966 | [0.2832;1.6487] | 0.0056 |
| SPARC | 0.7581 | [0.2042;1.3121] | 0.0073 |
| SPATA2 | 0.6988 | [0.3824;1.0153] | <0.0001 |
| SPATS2 | 1.4526 | [0.8422;2.0631] | <0.0001 |
| SPC24 | 1.0959 | [0.0891;2.1027] | 0.0329 |
| SPC25 | 1.0339 | [0.0072;2.0606] | 0.0484 |
| SPDL1 | 0.979 | [0.5020;1.4560] | <0.0001 |
| SPIDR | 0.7518 | [0.2635;1.2401] | 0.0025 |
| SPINT2 | 1.1684 | [0.2855;2.0512] | 0.0095 |
| SPIRE1 | 0.9778 | [0.2906;1.6649] | 0.0053 |
| SPP1 | 0.7288 | [0.4242;1.0333] | <0.0001 |
| SPPL3 | 1.4114 | [0.5462;2.2766] | 0.0014 |
| SPR | 1.6631 | [0.8526;2.4737] | <0.0001 |
| SPSB4 | 0.684 | [0.3170;1.0511] | 0.0003 |
| SPTLC3 | 0.3441 | [0.0169;0.6713] | 0.0393 |
| SQLE | 0.8819 | [0.5666;1.1971] | <0.0001 |
| SQSTM1 | 0.3338 | [0.0029;0.6646] | 0.048 |
| SRD5A1 | 1.8101 | [1.0450;2.5751] | <0.0001 |
| SREK1IP1 | 0.4673 | [0.1326;0.8019] | 0.0062 |
| SRGAP1 | 0.7263 | [0.1852;1.2674] | 0.0085 |
| SRSF12 | 0.6465 | [0.3036;0.9893] | 0.0002 |
| SSH1 | 0.7398 | [0.0128;1.4668] | 0.0461 |
| SSTR2 | 1.2762 | [0.9424;1.6100] | <0.0001 |
| SSX2IP | 1.3454 | [1.0101;1.6806] | <0.0001 |
| ST6GALNAC2 | 1.1112 | [0.3029;1.9195] | 0.0071 |
| ST7 | 0.9285 | [0.5251;1.3318] | <0.0001 |
| STAMBP | 0.7318 | [0.0286;1.4349] | 0.0414 |
| STAP2 | 2.1555 | [0.9589;3.3520] | 0.0004 |
| STAR | 1.4027 | [1.0741;1.7313] | <0.0001 |
| STARD5 | 0.3717 | [0.0437;0.6998] | 0.0264 |
| STAT1 | 1.3464 | [0.7781;1.9146] | <0.0001 |
| STC2 | 1.0444 | [0.7218;1.3670] | <0.0001 |
| STEAP1 | 0.6891 | [0.1162;1.2619] | 0.0184 |
| STEAP3 | 1.4442 | [0.4760;2.4124] | 0.0035 |
| STIL | 1.4441 | [0.6014;2.2868] | 0.0008 |
| STMN1 | 1.0751 | [0.7532;1.3971] | <0.0001 |
| STRA6 | 0.4837 | [0.1508;0.8166] | 0.0044 |
| STX3 | 0.4207 | [0.0903;0.7511] | 0.0126 |
| STX6 | 1.5974 | [0.8950;2.2998] | <0.0001 |
| SUCNR1 | 0.9507 | [0.6239;1.2775] | <0.0001 |
| SULF1 | 0.9866 | [0.6647;1.3085] | <0.0001 |
| SULT1E1 | 0.3419 | [0.0376;0.6462] | 0.0276 |
| SULT4A1 | 0.3551 | [0.0505;0.6598] | 0.0223 |
| SUPV3L1 | 0.7115 | [0.3979;1.0251] | <0.0001 |
| SUSD4 | 1.5728 | [0.7329;2.4128] | 0.0002 |
| SUV39H2 | 0.9227 | [0.2129;1.6324] | 0.0108 |
| SUZ12 | 0.6863 | [0.3459;1.0266] | <0.0001 |
| SYCE3 | 1.6154 | [0.4902;2.7405] | 0.0049 |
| SYNCRIP | 0.8282 | [0.2522;1.4042] | 0.0048 |
| SYNPO2 | 1.179 | [0.4128;1.9453] | 0.0026 |
| SYNPO2L | 0.3175 | [0.0150;0.6199] | 0.0396 |
| TAGLN2 | 0.6657 | [0.0953;1.2361] | 0.0222 |
| TARBP2 | 0.6392 | [0.3262;0.9522] | <0.0001 |
| TARS | 1.2648 | [0.9252;1.6045] | <0.0001 |
| TAX1BP3 | 0.7542 | [0.0680;1.4403] | 0.0312 |
| TBC1D24 | 0.9438 | [0.5855;1.3021] | <0.0001 |
| TBX3 | 0.7818 | [0.4729;1.0907] | <0.0001 |
| TCERG1L | 0.6826 | [0.3464;1.0188] | <0.0001 |
| TCF7L1 | 0.3809 | [0.0761;0.6857] | 0.0143 |
| TDO2 | 0.4708 | [0.1670;0.7746] | 0.0024 |
| TDRP | 0.9314 | [0.4349;1.4278] | 0.0002 |
| TEAD2 | 0.9672 | [0.4023;1.5321] | 0.0008 |
| TEAD4 | 1.6198 | [0.9309;2.3088] | <0.0001 |
| TENM3 | 1.9772 | [0.5195;3.4348] | 0.0078 |
| TEX101 | 0.8486 | [0.4947;1.2024] | <0.0001 |
| TEX11 | 0.8175 | [0.5088;1.1262] | <0.0001 |
| TEX19 | 0.7826 | [0.4225;1.1427] | <0.0001 |
| TEX30 | 0.6365 | [0.1866;1.0864] | 0.0056 |
| TFAP2A | 1.1193 | [0.3702;1.8683] | 0.0034 |
| TFAP2C | 0.8081 | [0.0537;1.5626] | 0.0358 |
| TFEC | 0.426 | [0.1171;0.7349] | 0.0069 |
| TFG | 1.3114 | [0.7365;1.8864] | <0.0001 |
| TFPI2 | 0.3322 | [0.0274;0.6369] | 0.0327 |
| TFRC | 1.1399 | [0.5016;1.7782] | 0.0005 |
| TGFB2 | 0.7541 | [0.4415;1.0666] | <0.0001 |
| TGFBI | 0.8358 | [0.5206;1.1510] | <0.0001 |
| TGIF2 | 1.0951 | [0.5136;1.6765] | 0.0002 |
| TGS1 | 0.69 | [0.3597;1.0203] | <0.0001 |
| THAP2 | 0.8402 | [0.4869;1.1935] | <0.0001 |
| THAP9 | 0.5352 | [0.0372;1.0332] | 0.0352 |
| THBS2 | 0.7133 | [0.3714;1.0553] | <0.0001 |
| THBS4 | 0.9561 | [0.6368;1.2755] | <0.0001 |
| THNSL1 | 0.5978 | [0.2869;0.9087] | 0.0002 |
| THOC3 | 1.1902 | [0.7815;1.5990] | <0.0001 |
| THSD7B | 0.5379 | [0.2013;0.8744] | 0.0017 |
| THUMPD3 | 0.5351 | [0.2155;0.8547] | 0.001 |
| THY1 | 0.7545 | [0.4427;1.0662] | <0.0001 |
| TIMELESS | 0.9853 | [0.2299;1.7406] | 0.0106 |
| TIMMDC1 | 1.885 | [0.9002;2.8699] | 0.0002 |
| TIPIN | 1.3315 | [0.5496;2.1134] | 0.0008 |
| TK1 | 1.4968 | [0.9424;2.0512] | <0.0001 |
| TKT | 0.4801 | [0.1686;0.7915] | 0.0025 |
| TLE1 | 0.6461 | [0.0443;1.2479] | 0.0354 |
| TLR3 | 1.1459 | [0.2953;1.9965] | 0.0083 |
| TLR5 | 0.6702 | [0.1601;1.1802] | 0.01 |
| TM4SF1 | 0.6363 | [0.0544;1.2181] | 0.0321 |
| TM4SF19 | 0.564 | [0.1215;1.0065] | 0.0125 |
| TM6SF1 | 0.3878 | [0.0800;0.6956] | 0.0135 |
| TM7SF3 | 1.791 | [1.4399;2.1420] | <0.0001 |
| TMED2 | 1.061 | [0.3741;1.7479] | 0.0025 |
| TMEM108 | 0.7363 | [0.3891;1.0834] | <0.0001 |
| TMEM117 | 0.4075 | [0.0642;0.7507] | 0.02 |
| TMEM128 | 0.754 | [0.4094;1.0986] | <0.0001 |
| TMEM132A | 1.273 | [0.4595;2.0864] | 0.0022 |
| TMEM144 | 0.382 | [0.0504;0.7137] | 0.024 |
| TMEM14A | 1.2079 | [0.8773;1.5384] | <0.0001 |
| TMEM150C | 0.8235 | [0.4701;1.1769] | <0.0001 |
| TMEM155 | 0.6437 | [0.3012;0.9862] | 0.0002 |
| TMEM171 | 0.4632 | [0.1143;0.8121] | 0.0093 |
| TMEM194A | 1.2863 | [0.8902;1.6825] | <0.0001 |
| TMEM200A | 1.0139 | [0.6704;1.3574] | <0.0001 |
| TMEM200C | 0.7388 | [0.3932;1.0844] | <0.0001 |
| TMEM237 | 1.2614 | [0.4914;2.0314] | 0.0013 |
| TMEM30A | 0.9606 | [0.4144;1.5068] | 0.0006 |
| TMEM33 | 1.472 | [1.1330;1.8109] | <0.0001 |
| TMEM38B | 0.9803 | [0.6345;1.3261] | <0.0001 |
| TMEM51 | 1.3599 | [0.3731;2.3467] | 0.0069 |
| TMEM54 | 1.0103 | [0.1516;1.8690] | 0.0211 |
| TMEM68 | 1.3396 | [0.6919;1.9874] | <0.0001 |
| TMEM97 | 0.9741 | [0.3919;1.5562] | 0.001 |
| TMPO | 1.4326 | [1.0951;1.7701] | <0.0001 |
| TMPRSS11A | 0.6212 | [0.1904;1.0520] | 0.0047 |
| TMPRSS13 | 0.401 | [0.0560;0.7461] | 0.0227 |
| TMTC2 | 0.8702 | [0.0093;1.7311] | 0.0476 |
| TMTC3 | 1.4622 | [0.6677;2.2568] | 0.0003 |
| TMTC4 | 0.5308 | [0.1822;0.8794] | 0.0028 |
| TNFAIP2 | 0.8039 | [0.4894;1.1183] | <0.0001 |
| TNFAIP3 | 1.0333 | [0.7170;1.3496] | <0.0001 |
| TNFAIP6 | 1.9755 | [1.6173;2.3337] | <0.0001 |
| TNFRSF10B | 1.4406 | [1.1060;1.7752] | <0.0001 |
| TNFRSF12A | 0.7776 | [0.1271;1.4282] | 0.0191 |
| TNFSF10 | 0.6039 | [0.0166;1.1912] | 0.0439 |
| TNFSF15 | 1.5104 | [1.1723;1.8485] | <0.0001 |
| TNFSF4 | 1.3769 | [0.7767;1.9770] | <0.0001 |
| TNFSF9 | 0.7867 | [0.0707;1.5027] | 0.0313 |
| TNIP3 | 0.7563 | [0.4423;1.0703] | <0.0001 |
| TNKS1BP1 | 1.6628 | [0.7355;2.5902] | 0.0004 |
| TNNT1 | 0.8551 | [0.5413;1.1689] | <0.0001 |
| TNNT2 | 0.7555 | [0.1899;1.3210] | 0.0088 |
| TOM1L1 | 1.2953 | [0.5821;2.0085] | 0.0004 |
| TOMM40 | 1.0147 | [0.6904;1.3390] | <0.0001 |
| TONSL | 0.9613 | [0.1148;1.8077] | 0.026 |
| TOP2A | 1.708 | [0.9260;2.4900] | <0.0001 |
| TOPBP1 | 0.9703 | [0.2105;1.7301] | 0.0123 |
| TOR3A | 0.3438 | [0.0323;0.6553] | 0.0305 |
| TP53 | 1.0469 | [0.4330;1.6608] | 0.0008 |
| TP53BP2 | 0.7626 | [0.2286;1.2967] | 0.0051 |
| TP63 | 1.0775 | [0.3425;1.8125] | 0.0041 |
| TPBG | 0.9969 | [0.2946;1.6992] | 0.0054 |
| TPMT | 0.8477 | [0.2003;1.4950] | 0.0103 |
| TPRA1 | 0.4422 | [0.1064;0.7780] | 0.0098 |
| TPRKB | 0.8052 | [0.4625;1.1479] | <0.0001 |
| TPX2 | 1.5144 | [0.7751;2.2536] | <0.0001 |
| TRAF2 | 0.522 | [0.1870;0.8569] | 0.0023 |
| TRAM2 | 0.509 | [0.1995;0.8184] | 0.0013 |
| TRAP1 | 1.0752 | [0.4359;1.7144] | 0.001 |
| TREM1 | 0.5384 | [0.2318;0.8451] | 0.0006 |
| TREM2 | 0.9602 | [0.2524;1.6680] | 0.0078 |
| TRERF1 | 0.3717 | [0.0547;0.6886] | 0.0215 |
| TRIAP1 | 1.3632 | [1.0032;1.7232] | <0.0001 |
| TRIM16L | 1.6797 | [0.4559;2.9035] | 0.0071 |
| TRIM24 | 1.1014 | [0.5173;1.6856] | 0.0002 |
| TRIM33 | 0.4105 | [0.1013;0.7198] | 0.0093 |
| TRIM43 | 0.5624 | [0.1735;0.9512] | 0.0046 |
| TRIM47 | 0.862 | [0.0950;1.6291] | 0.0276 |
| TRIM5 | 0.7866 | [0.0254;1.5479] | 0.0428 |
| TRIP13 | 0.8404 | [0.1341;1.5466] | 0.0197 |
| TRIP6 | 1.5496 | [0.8065;2.2927] | <0.0001 |
| TRIT1 | 0.5601 | [0.2520;0.8683] | 0.0004 |
| TRNT1 | 0.413 | [0.0985;0.7276] | 0.0101 |
| TROAP | 0.897 | [0.1656;1.6284] | 0.0162 |
| TRPA1 | 0.6525 | [0.3473;0.9577] | <0.0001 |
| TRPS1 | 0.3878 | [0.0813;0.6942] | 0.0131 |
| TRUB2 | 0.7282 | [0.0939;1.3624] | 0.0244 |
| TSGA13 | 0.3491 | [0.0180;0.6802] | 0.0388 |
| TSHZ3 | 0.4132 | [0.0658;0.7606] | 0.0198 |
| TSKU | 1.0064 | [0.0319;1.9808] | 0.043 |
| TSPAN15 | 0.9355 | [0.3638;1.5072] | 0.0013 |
| TSPAN17 | 1.5468 | [0.5442;2.5494] | 0.0025 |
| TSPAN9 | 1.2938 | [0.5144;2.0731] | 0.0011 |
| TSPEAR | 0.957 | [0.2217;1.6922] | 0.0107 |
| TTC23 | 1.0256 | [0.4752;1.5760] | 0.0003 |
| TTK | 1.4452 | [1.1039;1.7865] | <0.0001 |
| TUBA1B | 1.1238 | [0.3958;1.8519] | 0.0025 |
| TUBA1C | 1.5978 | [0.7746;2.4209] | 0.0001 |
| TUBB | 0.8763 | [0.2799;1.4727] | 0.004 |
| TUBG1 | 0.8293 | [0.1963;1.4623] | 0.0102 |
| TUFT1 | 1.4237 | [0.5847;2.2628] | 0.0009 |
| TUSC1 | 0.7363 | [0.0041;1.4685] | 0.0487 |
| TUSC3 | 0.5498 | [0.2434;0.8562] | 0.0004 |
| TWF1 | 1.3141 | [0.5927;2.0355] | 0.0004 |
| TWSG1 | 1.2134 | [0.8948;1.5320] | <0.0001 |
| TXN | 1.3095 | [0.2675;2.3515] | 0.0138 |
| TXNDC17 | 1.0078 | [0.0531;1.9624] | 0.0385 |
| TXNRD1 | 0.5414 | [0.2147;0.8680] | 0.0012 |
| TYK2 | 0.8425 | [0.5004;1.1847] | <0.0001 |
| TYMP | 0.8504 | [0.5136;1.1873] | <0.0001 |
| TYMS | 1.4676 | [0.8623;2.0729] | <0.0001 |
| U2SURP | 1.3528 | [0.6807;2.0249] | <0.0001 |
| UACA | 0.3707 | [0.0591;0.6822] | 0.0197 |
| UBD | 0.8696 | [0.1747;1.5645] | 0.0142 |
| UBE2C | 0.9265 | [0.3761;1.4768] | 0.001 |
| UBE2E3 | 1.065 | [0.7396;1.3903] | <0.0001 |
| UBE2Q1 | 0.579 | [0.2395;0.9186] | 0.0008 |
| UBE2QL1 | 0.9685 | [0.6128;1.3243] | <0.0001 |
| UBE2S | 0.9443 | [0.3633;1.5253] | 0.0014 |
| UBE2T | 1.5733 | [0.6746;2.4720] | 0.0006 |
| UBE2V2 | 0.997 | [0.6810;1.3131] | <0.0001 |
| UBE2Z | 1.0321 | [0.3561;1.7081] | 0.0028 |
| UBFD1 | 1.0004 | [0.2307;1.7701] | 0.0109 |
| UBQLN4 | 0.7328 | [0.0907;1.3749] | 0.0253 |
| UGGT2 | 0.848 | [0.5092;1.1869] | <0.0001 |
| UGT2A1 | 0.4407 | [0.0424;0.8390] | 0.0301 |
| UHRF1 | 0.9964 | [0.4303;1.5625] | 0.0006 |
| ULBP2 | 1.3693 | [0.5903;2.1482] | 0.0006 |
| UMODL1 | 0.4191 | [0.0799;0.7582] | 0.0154 |
| UNC5B | 1.0836 | [0.3127;1.8546] | 0.0059 |
| UNC79 | 0.4273 | [0.0682;0.7863] | 0.0197 |
| UNG | 1.2952 | [0.9644;1.6260] | <0.0001 |
| UPK3B | 0.4732 | [0.1390;0.8075] | 0.0055 |
| UPP1 | 0.42 | [0.1112;0.7288] | 0.0077 |
| URB2 | 0.8109 | [0.1144;1.5075] | 0.0225 |
| USP18 | 1.6961 | [1.0442;2.3481] | <0.0001 |
| USP31 | 1.3757 | [0.9962;1.7551] | <0.0001 |
| USP41 | 1.4097 | [0.8329;1.9865] | <0.0001 |
| UXS1 | 1.3847 | [1.0530;1.7165] | <0.0001 |
| VAMP7 | 0.5209 | [0.1914;0.8504] | 0.0019 |
| VANGL2 | 1.3668 | [0.5826;2.1510] | 0.0006 |
| VASH2 | 1.4152 | [1.0519;1.7784] | <0.0001 |
| VCAM1 | 0.5229 | [0.2185;0.8272] | 0.0008 |
| VCAN | 0.9947 | [0.6465;1.3428] | <0.0001 |
| VCL | 0.8056 | [0.2984;1.3127] | 0.0019 |
| VDAC1 | 0.9473 | [0.6309;1.2637] | <0.0001 |
| VDR | 0.9475 | [0.2561;1.6389] | 0.0072 |
| VEGFA | 1.0339 | [0.6882;1.3796] | <0.0001 |
| VIL1 | 0.6224 | [0.3150;0.9297] | <0.0001 |
| VLDLR | 0.7999 | [0.1876;1.4121] | 0.0104 |
| VPS29 | 0.3751 | [0.0544;0.6958] | 0.0219 |
| VPS54 | 1.2343 | [0.5584;1.9103] | 0.0003 |
| VRK2 | 1.7537 | [1.4032;2.1042] | <0.0001 |
| VSNL1 | 1.0984 | [0.5376;1.6591] | 0.0001 |
| WBP5 | 1.3163 | [0.4738;2.1588] | 0.0022 |
| WDHD1 | 1.2687 | [0.4851;2.0523] | 0.0015 |
| WDR3 | 0.9336 | [0.5911;1.2760] | <0.0001 |
| WDR41 | 1.0315 | [0.5025;1.5606] | 0.0001 |
| WDR47 | 1.0043 | [0.6625;1.3460] | <0.0001 |
| WDR61 | 0.7105 | [0.3546;1.0664] | <0.0001 |
| WDSUB1 | 0.8015 | [0.1287;1.4743] | 0.0195 |
| WEE1 | 0.726 | [0.0718;1.3802] | 0.0296 |
| WFDC5 | 1.0849 | [0.4068;1.7631] | 0.0017 |
| WIZ | 0.9854 | [0.1754;1.7954] | 0.0171 |
| WNK2 | 1.9003 | [0.9123;2.8884] | 0.0002 |
| WNT10B | 0.7472 | [0.4334;1.0611] | <0.0001 |
| WNT2 | 0.6043 | [0.2948;0.9137] | 0.0001 |
| WNT4 | 0.7917 | [0.1172;1.4662] | 0.0214 |
| WNT5A | 1.3006 | [0.6466;1.9547] | <0.0001 |
| WNT7B | 0.6402 | [0.0933;1.1871] | 0.0218 |
| WRAP73 | 0.9162 | [0.5111;1.3213] | <0.0001 |
| WRN | 0.9092 | [0.5873;1.2311] | <0.0001 |
| WSB2 | 1.0938 | [0.5289;1.6587] | 0.0001 |
| WWC2 | 0.8965 | [0.1814;1.6115] | 0.014 |
| XAF1 | 0.9763 | [0.6291;1.3235] | <0.0001 |
| XG | 1.2137 | [0.8805;1.5469] | <0.0001 |
| XIRP1 | 0.5011 | [0.1591;0.8431] | 0.0041 |
| XKR4 | 0.4974 | [0.1600;0.8347] | 0.0039 |
| XPO1 | 0.9405 | [0.4023;1.4787] | 0.0006 |
| XPOT | 1.3224 | [0.6507;1.9940] | 0.0001 |
| XPR1 | 0.4292 | [0.1121;0.7464] | 0.008 |
| YBX3 | 1.6084 | [0.5086;2.7083] | 0.0042 |
| YES1 | 1.4151 | [0.6409;2.1894] | 0.0003 |
| ZBED1 | 0.6244 | [0.3168;0.9320] | <0.0001 |
| ZBED3 | 1.2631 | [0.9349;1.5913] | <0.0001 |
| ZBED8 | 0.5384 | [0.0593;1.0175] | 0.0276 |
| ZBTB18 | 0.6114 | [0.1630;1.0597] | 0.0075 |
| ZC3H12C | 0.9244 | [0.2679;1.5808] | 0.0058 |
| ZC3H7B | 0.7894 | [0.1967;1.3822] | 0.009 |
| ZCCHC14 | 0.7441 | [0.0115;1.4766] | 0.0465 |
| ZDBF2 | 0.9211 | [0.5694;1.2728] | <0.0001 |
| ZDHHC9 | 0.6685 | [0.1312;1.2057] | 0.0147 |
| ZFP30 | 0.762 | [0.4221;1.1020] | <0.0001 |
| ZIC2 | 1.6557 | [1.2954;2.0159] | <0.0001 |
| ZMYND19 | 0.4787 | [0.1563;0.8012] | 0.0036 |
| ZNF112 | 1.3212 | [0.1720;2.4705] | 0.0242 |
| ZNF121 | 0.9498 | [0.0323;1.8673] | 0.0425 |
| ZNF205 | 0.6838 | [0.1027;1.2648] | 0.0211 |
| ZNF223 | 0.642 | [0.3087;0.9753] | 0.0002 |
| ZNF267 | 0.5341 | [0.2213;0.8469] | 0.0008 |
| ZNF280C | 0.6925 | [0.3391;1.0458] | 0.0001 |
| ZNF281 | 0.846 | [0.1505;1.5416] | 0.0171 |
| ZNF282 | 0.6099 | [0.1197;1.1000] | 0.0147 |
| ZNF354A | 0.7677 | [0.2414;1.2940] | 0.0042 |
| ZNF367 | 0.9231 | [0.1856;1.6606] | 0.0142 |
| ZNF385C | 0.9429 | [0.1220;1.7638] | 0.0244 |
| ZNF462 | 1.1408 | [0.4273;1.8543] | 0.0017 |
| ZNF473 | 0.8671 | [0.2131;1.5212] | 0.0094 |
| ZNF488 | 1.3397 | [0.4749;2.2045] | 0.0024 |
| ZNF503 | 1.0392 | [0.2974;1.7810] | 0.006 |
| ZNF516 | 0.7167 | [0.1109;1.3226] | 0.0204 |
| ZNF532 | 0.7604 | [0.4481;1.0726] | <0.0001 |
| ZNF556 | 0.5164 | [0.2112;0.8215] | 0.0009 |
| ZNF576 | 0.5441 | [0.2344;0.8538] | 0.0006 |
| ZNF584 | 1.022 | [0.2416;1.8024] | 0.0103 |
| ZNF609 | 0.4035 | [0.0703;0.7366] | 0.0176 |
| ZNF618 | 1.6782 | [0.8448;2.5117] | <0.0001 |
| ZNF629 | 1.0858 | [0.1075;2.0641] | 0.0296 |
| ZNF664 | 1.1417 | [0.4908;1.7926] | 0.0006 |
| ZNF670 | 0.3786 | [0.0350;0.7222] | 0.0308 |
| ZNF682 | 0.4166 | [0.0854;0.7477] | 0.0137 |
| ZNF683 | 1.2183 | [0.4621;1.9745] | 0.0016 |
| ZNF697 | 1.2112 | [0.4020;2.0204] | 0.0033 |
| ZNF713 | 0.6621 | [0.0720;1.2523] | 0.0279 |
| ZNF74 | 1.1819 | [0.8635;1.5003] | <0.0001 |
| ZNF768 | 0.8568 | [0.3956;1.3181] | 0.0003 |
| ZNF833P | 1.6672 | [0.8104;2.5239] | 0.0001 |
| ZNF84 | 0.9607 | [0.2651;1.6563] | 0.0068 |
| ZNRF1 | 1.2739 | [0.4719;2.0759] | 0.0019 |
| ZNRF3 | 1.6735 | [1.0232;2.3238] | <0.0001 |
| ZP3 | 0.6395 | [0.1868;1.0921] | 0.0056 |
| ZP4 | 0.6972 | [0.3584;1.0360] | <0.0001 |
| ZRANB3 | 0.7899 | [0.0418;1.5380] | 0.0385 |
| ZSCAN12 | 0.3403 | [0.0138;0.6667] | 0.0411 |
| ZWILCH | 1.3775 | [0.7106;2.0444] | <0.0001 |
| ZWINT | 1.2195 | [0.2828;2.1563] | 0.0107 |
